# ABIN-1 is a key regulator in RIPK1-dependent apoptosis (RDA) and necroptosis, and ABIN-1 deficiency potentiates necroptosis-based cancer therapy in colorectal cancer

**DOI:** 10.1038/s41419-021-03427-y

**Published:** 2021-02-01

**Authors:** Jiali Cai, Die Hu, Judy Sakya, Tao Sun, Daoyong Wang, Lin Wang, Xiaohua Mao, Zhenyi Su

**Affiliations:** 1 Department of Biochemistry and Molecular Biology, School of Medicine, Southeast University, 210009 Nanjing, Jiangsu China; 2 School of Life Science and Technology, Southeast University, 210009 Nanjing, Jiangsu China; 3 School of Medicine, Texas Tech University Health Sciences Center, 3601 4th St, Lubbock, TX 79430 USA; 4 School of Medicine, Southeast University, 210009 Nanjing, Jiangsu China; 5 Institute for Cancer Genetics, Department of Pathology and Cell Biology, Herbert Irving Comprehensive Cancer Center, College of Physicians & Surgeons, Columbia University, 1130 Nicholas Ave, New York, NY 10032 USA

**Keywords:** Cancer, Cell death

## Abstract

ABIN-1, also called TNIP1, is an ubiquitin-binding protein that serves an important role in suppressing RIPK1-independent apoptosis, necroptosis, and NF-κB activation. However, the involvement of ABIN-1 in the regulation of RIPK1-dependent apoptosis (RDA) is unknown. In this study, we found that poly(I:C) + TAK1 inhibitor 5Z-7-oxozeaenol (P5) concurrently induces RDA and necroptosis in *Abin-1*^−/−^, but not in *Abin-1*^+/+^ mouse embryonic fibroblasts (MEFs). Upon P5 stimulation, cells initially die by necroptosis and subsequently by RDA. Furthermore, we explored the therapeutic effect of ABIN-1 deficiency in necroptosis-based cancer therapy in colorectal cancer (CRC). We found that poly(I:C) + 5Z-7-oxozeaenol + IDN-6556 (P5I) yields a robust pro-necroptosis response, and ABIN-1 deficiency additionally enhances this P5I-induced necroptosis. Moreover, phase I/II cIAP inhibitor birinapant with clinical caspase inhibitor IDN-6556 (BI) alone and 5-fluorouracil with IDN-6556 (FI) alone are sufficient to induce necroptotic cell death in CRC cells by promoting auto-secretion of tumor necrosis factor (TNF); ABIN-1 deficiency amplifies the BI- or FI-induced necroptosis. Two independent xenograft experiments using HT-29 or COLO205 cells show that both BI and P5I remarkably inhibit tumor growth via necroptosis activation. For poly(I:C)-induced cell death, the sensitizing effect of ABIN-1 deficiency on cell death may be attributed to increased expression of TLR3. In TNF-induced necroptosis, ABIN-1 deficiency increases TNF-induced RIPK1 polyubiquitination by reducing the recruitment of ubiquitin-editing enzyme A20 to the TNFR1 signaling complex and induces more TNF secretion in CRC cells upon pro-necroptosis stimulation. With this combined data, ABIN-1 deficiency promotes greater sensitization of CRC cells to necroptosis.

## Introduction

Tumor necrosis factor (TNF) stimulation can promote three different cell death pathways, including receptor-interacting serine-threonine kinase (RIPK) 1-independent apoptosis, RIPK1-dependent apoptosis (RDA), and necroptosis^1^. When cells are treated with TNF and cycloheximide, cells undergo RIPK1-independent apoptosis. Under inhibitor of apoptosis proteins 1 (IAP1)- and IAP2-deficient conditions, or NF-κB essential modulator (NEMO), IκB kinase (IKK)- or TGF-β-activated kinase 1 (TAK1)-deficient conditions, an activated, detergent-insoluble ubiquitylated RIPK1 species (iuRIPK1) promotes RIPK1 dimerization and RDA^2–4^. In the siRNA screening by Amin et al., never-in-mitosis A-related kinase 1 (NEK1), leucine-rich repeat kinase-2 (LRRK2), and the E3 ligases APC11 and c-Cbl are also involved in the regulation of RDA^3^. Furthermore, caspase-8 can block necroptosis by proteolytically cleaving RIPK1 at site Asp325 and suppressing RIPK1 kinase activation^5,6^. Therefore, inhibition of caspase-8 activation by zVAD.fmk or IDN-6556 will promote necroptosis. The RIPK1-RIPK3- mixed lineage kinase domain-like (MLKL) complex, also called the “necrosome”, is assembled during necroptosis, and the MLKL-dependent cell membrane permeabilization eventually leads to cell necrosis^7–9^. RIPK1 is modified by several types of ubiquitination proteins including M1, K11, K48, and K63 that all play an important role in regulating RIPK1 activation^10–12^.

In addition to TNF-induced necroptosis, another common necroptotic cell death is Toll-like receptor 3 (TLR3)-induced necroptosis triggered by poly(I:C) and caspase inhibitors. Specifically, the engagement of TLR3 induces the oligomerization of the adaptor protein TRIF that in turn recruits cIAPs, caspase-8/FADD, and RIPK1 leading to either apoptosis or necroptosis^13^ If caspase-8 is inhibited by caspase inhibitors, RIPK1 recruits RIPK3 and MLKL to necroptosis; however, if caspase-8 is active, cells mostly undergo apoptosis^13,14^. Currently, it is unclear if suppression of TAK1 can promote RDA upon poly(I:C) stimulation.

A20-binding inhibitor of NF-κB1 (ABIN-1), also called TNIP1, is an ubiquitin-binding protein with a UBAN domain, which serves an important role in suppressing apoptosis, necroptosis, and NF-κB activation^15,16^. ABIN-1 deficiency also enhances innate immunity and antiviral responses^17^. Specific haplotypes or dysfunction in *Abin-1* increase the risk of suffering autoimmune diseases^18^. Previously, we have proven that ABIN-1 is recruited to the TNFR1 signaling complex (TNF-RSC) in an M1 ubiquitination-dependent manner to facilitate the recruitment of the ubiquitin-editing enzyme A20. A20 functions as a K63 deubiquitinase of RIPK1 and deficiency of A20 thus promotes necroptosis by activating RIPK1 and its pro-necroptotic partner RIPK3^16,19^. *Abin-1*^*−/*−^ mice die at embryonic day 18.5 from liver damage that can be rescued by *Ripk1*^D138N^ kinase-dead mutation or by *Ripk3* deficiency^16^. ABIN-1 deficiency can transform TNF + cyclohyximide (TC)-induced RIPK1-independent apoptosis into both RIPK1-independent apoptosis and necroptosis in mouse embryonic fibroblasts (MEFs)^16^, However, it is unknown whether ABIN-1 deficiency also participates in the regulation of RDA. Moreover, further investigation is necessary to understand whether targeting ABIN-1 can be applied to improve necroptosis-based cancer therapy.

Necroptosis-based cancer therapy is an alternative option when pro-apoptosis chemotherapy fails due to drug resistance. Many proposed mechanisms of cancer therapy failure are disrupted apoptosis machinery, strengthened prosurvival signals, increased expression of therapeutic targets, activation of compensatory pathways, high molecular heterogeneity in tumor cells, upregulation of drug transporters, and multidrug resistance^20,21^. Among these reasons, disrupted apoptosis machinery due to caspase inhibition and the defect is a critical factor in intrinsic and acquired chemotherapy drug resistance. Therefore, necroptosis may kill cancer cells with defective and inhibited caspases^22^. In addition, necroptotic cancer cells release many damage-associated molecular patterns (DAMPs) and tumor antigens to activate dendritic cells, which in turn activates CD8 + T cells and antitumor immunity^23^. Notably, RIPK1 and NF-κB signaling in necroptotic cells is critical for CD8 + T-cell cross-priming^24^. In the past few years, some studies have illustrated the potential therapeutic effect of necroptosis-based cancer therapy in tumor models^22,25^. A study by Xie et al. showed that inhibition of Aurora kinase A (a negative regulator of RIPK1-RIPK3-MLKL necrosome) by CCT137690-induced necroptosis and suppressed the growth and progression of both subcutaneous and orthotopic pancreatic tumors in mice^26^. 5-FU with pan-caspase inhibitor IDN-7314-induced TNF-dependent necroptosis in colon cancer cells showed a better suppressive effect in tumor growth in HT-29 xenograft model compared to 5-fluorouracil alone. Moreover, around 50% of tumor samples from colon cancer patients were more sensitive to necroptosis inducer 5-FU + zVAD than apoptosis inducer 5-FU^27^. Poly(I:C) + zVAD also served as a necroptosis inducer and inhibit tumor growth in colon cancer xenograft model^28^.

Considering colorectal cancer (CRC) cells are more responsive to pro-necroptosis stimuli compared with other cancer types, it would be interesting to explore if ABIN-1 deficiency improves the necroptosis-based cancer therapy in the CRC xenograft model. Currently, 5-fluorouracil (5-FU)-based chemotherapy is still the first-line therapy for CRC. Despite aggressive treatment with 5-FU-based chemotherapy and immunotherapy, the 5-year survival rate for metastatic CRC is only ~14%^29^. The main reason is the occurrence of drug resistance, and ~50% of metastatic CRC patients show resistance to 5-FU-based chemotherapy^30^. Triggering necroptosis has been proposed as an alternative method of circumventing this problem.

In this study, we found that poly(I:C) + TAK1 inhibitor 5Z-7-oxozeaenol (P5) concurrently induces RDA and necroptosis in *Abin-1*^*−/−*^ mouse embryonic fibroblasts (MEFs), but not in wild-type MEFs. Upon P5 stimulation, cells die by necroptosis in the early stage and die by RDA in the late stage. We further explored if ABIN-1 deficiency sensitizes CRC cells to necroptosis-based cancer therapy. We found that deficiency of ABIN-1 sensitizes CRC cells to both TNF-induced necroptosis and poly(I:C)-induced necroptosis in vitro and in vivo. Two independent xenograft experiments using HT-29 or COLO205 cells show that phase I/II cIAP inhibitor birinapant + clinical caspase inhibitor IDN-6556 (BI) or poly(I:C) + 5Z-7-oxozeaenol+IDN-6556 (P5I) remarkably inhibits tumor growth via necroptosis activation. For poly(I:C)-induced cell death, the sensitizing effect of ABIN-1 deficiency on cell death may be attributed to increased expression of TLR3. Furthermore, for TNF-induced necroptosis, sensitization may be due to increases in TNF-induced RIPK1 polyubiquitination by reducing the recruitment of ubiquitin-editing enzyme A20 to the TNFR1 signaling complex and increase in TNF secretion in CRC cells upon pro-necroptosis stimulation.

## Materials and methods

### Reagents

Human recombinant TNFα (R&D Systems, Cat#210-TA-005); Recombinant mouse mTNFα (Cell sciences, Cat# CRT192C); Cycloheximide (Sigma, Cat# C-6255); SM-164 (Selleckchem, custom-synthesized); Birinapant (Selleck Chemicals, Cat# S7015); zVAD (Selleck Chemicals, Cat# S8102); Nec-1s (7-Cl-O-Nec-1) (Selleck Chemicals, Cat# S8641); GSK-872 (Sigma, Cat# 5303890001); Necrosulfonamide (Sigma, 480073); LPS (Sigma, Cat# L4391); IDN-6556 (Selleck Chemicals, Cat# S7775); Poly(I:C) (InvivoGen, Cat# tlrl-pic-5); 5Z-7-Oxozeaenol (Sigma, Cat# O9890); 5-fluorouracil (Selleck Chemicals, Cat# S1209); Necrosulfonamide (Selleck Chemicals, Cat# S825); and ELISA Kit for Human TNFα (BioLegend, Cat# 430206).

### Cell lines, cell culture, siRNA, and lentivirus shRNA

CRC cell lines HT-29, COLO205, Caco-2, and HCT116 were from the Cell Bank of the Chinese Academy of Sciences (Shanghai, China). COLO205 was cultured in RPMI-1640 supplemented with 10% FBS, penicillin, and streptomycin. HT-29, HCT116, and Caco-2 were cultured in DMEM supplemented with 10% FBS, penicillin, and streptomycin. All cell lines were authenticated by quantitative PCR (qPCR) or western blot assay and tested for mycoplasma contamination.

Knockdowns were generated using Lipofectamine™ RNAiMAX (Thermo Fisher Scientific, Cat# 13778030) and the following siRNA sequences: human *Tlr3*: 5′- CAGCAUCUGUCUUUAAUAATT-3′; human *Tak1*: 5′-UGGCUUAUCUUACACUGGA-3′; human *Abin-1* siRNA-1: 5′-CAGGAGAGCGUUACCAUGUGGTT-3′; human *Abin-1* siRNA-2: 5′-GAAUACACCUGGCGUCUACTT-3′; mouse *Ripk3* siRNA: 5′-CGACGAUGUCUUCUGUCA-3′; mouse *Mlkl*: 5′-GAGAUCCAGUUCAACGAUA-3′; mouse *Rig-I*: 5′-CCGGACTTCGAACACGTTTAA-3′; mouse *Mda5*: 5′-GAACGUAGACGACAUAUUA-3′; mouse *Tlr3*: 5′-AAGGAUGUUUUCGGGCCGCCU-3′; Control siRNA: 5′-UUCUCCGAACGUGUCACGUTT-3′.

Lentivirus-packaged NC shRNA and Abin-1 shRNA were made by Shanghai Genechem Co., Ltd. (China). *Abin-1* shRNA sequence: 5′-GAAUACACCUGGCGUCUACTT-3′.

### Generation and immortalization of MEFs

*Abin-1*^*+/–*^ mice were bred with *Abin-1*^*+/–*^*Ripk1*^D138N^ or *Abin-1*^*+/–*^*Ripk3*^*–/–*^ mice, and pregnancy was terminated at the E11-13 stage. Embryos were homogenized individually and treated with trypsin/EDTA, sieved through a 70-µm filter and primary MEFs were cultured in high-glucose DMEM supplemented with 15% FBS, nonessential amino acids, sodium pyruvate, penicillin, streptomycin, and amphotericin B. At passages 4–6, primary MEFs were immortalized by transfection with SV40 small + large T antigen-expressing plasmid (Addgene, Cat# 22298) and keeping culturing for a few generations.

### Cell death assay

Cell death was determined using ToxiLight Non-destructive Cytotoxicity BioAssay Kit (Lonza, Cat# LT07-217). All experiments were conducted on 96-well plates using three biological replicates. Data were collected using Infinite F200 PRO Microplate Reader (Tecan, Swiss).

### qPCR analysis and primers

The total RNA was extracted using RNAiso Plus (Trizol) (Takara, Cat# 9108). Reverse transcription was performed with PrimeScript™ RT Reagent Kit with gDNA Eraser (Takara, Cat# RR047A). Real-time PCR primers were designed with the Primer 5.0 software, and the sequences were (5′−3′): TLR3: TTGCCTTGTATCTACTTTTGGGG (F), GCGGCTGGTAATCTTCTGAGTT (R); RIG-I: CAGACAGATCCGAGACACTA (F), TGCAAGACCTTTGGCCAGTT (R); MDA5: CGATCCGAATGATTGATGCA (F), AGTTGGTCATTGCAACTGCT (R); RIPK3: GTGCTACCTACACAGCTTGAAC (F), CCCTCCCTGAAACGTGGAC (R); MLKL: TTAGGCCAGCTCATCTATGAACA (F), TGCACACGGTTTCCTAGACG (R); and β-actin: GTCATTCCAAATATGAGATGCGT (F), GCTATCACCTCCCCTGTGTG (R). qPCR was performed on StepOnePlus™ Real-Time PCR System (Applied Biosystems) using SYBR^®^ Green Master Mix reagent (Applied Biosystems, Cat# 4309155).

### Antibodies and immunoprecipitation (IP)

The following antibodies were used: ABIN-1 (Proteintech, Cat# 15104-1-AP; Ubiquigent, Cat# 68-0001-100), TNFα (Abcam, Cat# ab183218), TLR3 (Cell Signaling, Cat# 6961), A20 (Cell Signaling, Cat# 5630), RIPK1 (Cell Signaling, Cat# D94C12); Phospho-RIPK1 (Ser166) (Cell Signaling, Cat# 65746); human phospho-MLKL (S358) (Abcam, Cat# ab187091); human MLKL (Abcam, Cat# ab183770); mouse phospho-MLKL (Abcam, Cat# ab196436); mouse MLKL (Sigma, Cat# SAB1302339); caspase-8 (Enzo, Cat# ALX-804-447-C100); PARP-1 (Cell Signaling, Cat# 9542); cleaved caspase-3 (Cell Signaling, Cat# 9661); TNF neutralization antibody (R&D Systems, Cat#MAB4101); β-actin (Proteintech Cat# 20536-1-AP); TNFR1 (Santa Cruz Biotechnology, Cat# sc-8436); cIAP1 (Cell Signaling, Cat# 7065). For complex I IP, cells were harvested in 1% NP40 lysis buffer (1% NP40, 150 mM NaCl, 50 mM Tris-HCl, 1 mM EDTA) and incubated overnight with 2 μg of TNFR1 antibody followed by 2-h incubation with Protein A/G ultra-link resin (Thermo Scientific, Cat# 53133). Then, beads were washed, and proteins were eluted with 2× SDS-PAGE gel electrophoresis loading buffer. For K63 ubiquitin IP, cells were lysed with 6 M urea lysis buffer (6 M urea, 20 mM Tris-HCl, pH 7.5, 135 mM NaCl, 1.5 mM MgCl_2_, 1 mM EGTA, 1% Triton X-100, complete protease inhibitor cocktail (Roche), 20 mM NEM, 1 mM PMSF, 5 mM Na pyrophosphate, 2 mM Na_3_VO_4_, 50 mM NaF, 5 mM sodium glycerophosphate) and centrifuged at full speed for 30 min in 4 °C. Supernatants were diluted with lysis buffer (without urea) to bring urea concentration to 3 M, and 2–3 μg chain-specific antibody was added and incubated at 4 °C overnight followed by 4-h incubation with Protein A agarose resin (Pierce, Cat# 20333). Beads were washed and proteins were eluted with 2× SDS-PAGE gel electrophoresis loading buffer.

### Xenograft experiment

HT-29 or COLO205 cells were transduced with lentivirus-based control shRNA (NC shRNA) or *Abin-1* shRNA and screened with puromycin for 5 days to establish stable knockdown pool cells. NC shRNA cells and *Abin-1* shRNA cells (5 × 10^6^) were subcutaneously injected into the left and right flank of nude mice (BALB/c nude mice, 6 weeks of age, from Experimental Animal Center of Yangzhou University, China), respectively. When tumors grew to 100 mm^3^, mice were randomly divided into different groups (2–5 mice each group) and given an intraperitoneal injection with indicated drugs every 3 days. Tumor volumes were measured every 2 or 3 days, and mice were sacrificed 14–18 days after the first drug injection, and tumors were isolated for imaging, weighing, western blot, and immunohistochemical staining. Birinapant, 2.5 mg/kg; IDN-6556, 1.25 mg/kg; poly(I:C), 5 mg/kg; 5Z-7-oxozeaenol, 0.6 mg/kg. Birinapant and IDN-6556 were dissolved in 15% captisol. All animals were housed in microisolator cages, with autoclaved food and bedding to minimize exposure to viral and microbial pathogens, and all procedures were approved by the Institutional Animal Care and Use Committee.

### H&E staining

Collected tumor tissues were fixed in 4% PFA and processed for paraffin embedding. The 6-μm-thick histological sections were stained with hematoxylin and eosin by the Wuhan Servicebio Technology Co., Ltd. (Wuhan, China). Images were taken with Olympus BX53 microscope and analyzed using MetaMorph image acquisition software.

### Statistics

Data were expressed as means ± standard error of the mean (S.E.M.) and analyzed by two-tailed *t* tests. Differences were considered statistically significant if *P < 0.05, **P < 0.01, or ***P < 0.001. The experiments were done at least three times in triplicate unless otherwise stated.

## Results

### Poly(I:C) + TAK1 inhibitor 5Z-7-oxozeaenol (5Z-7) concurrently induces RIPK1 kinase-dependent apoptosis and necroptosis in ABIN-1-deficient MEFs, while poly(I:C) + 5Z-7 + zVAD induces necroptosis

**Fig. 1:**
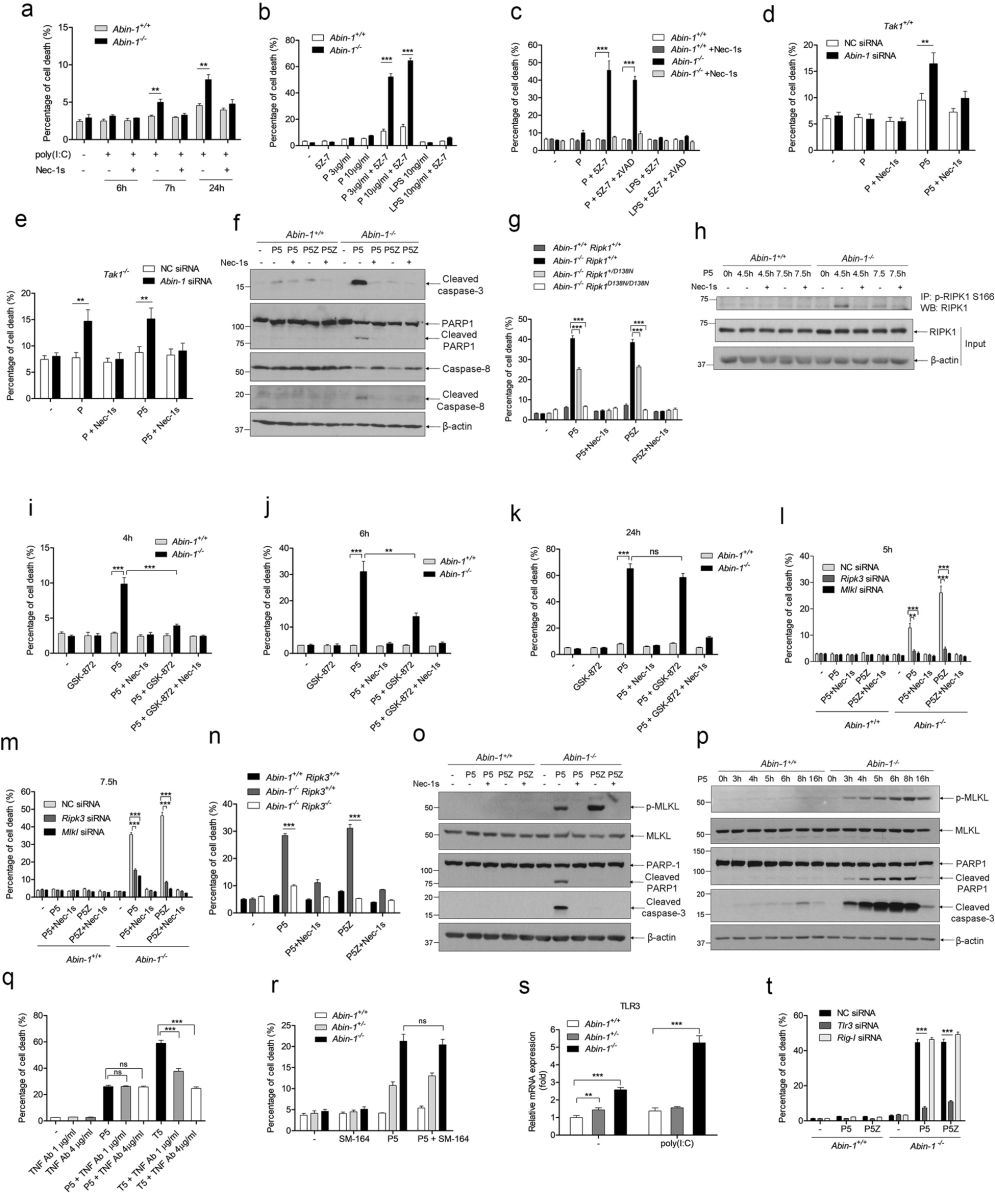
Poly(I:C) + 5Z-7-oxozeaenol concurrently induces RIPK1 kinase-dependent apoptosis and necroptosis in ABIN-1 deficient MEFs, while poly(I:C) + 5Z-7-oxozeaenol + zVAD induces necroptosis. **a** *Abin-1*^*+/+*^ MEFs and *Abin-1*^*−/−*^ MEFs were treated with 10 μg/ml poly(I:C) for 6 h, 7 h, or 24 h in presence or absence of necrostatin-1 (Nec-1s) and then the supernatant was subjected for ToxiLight assay. **b** *Abin-1*^*+/+*^ MEFs and *Abin-1*^*−/*^− MEFs were treated with 5Z-7-oxozeaenol (5Z-7), 3 μg/ml or 10 μg/ml poly(I:C) (P), 10 ng/ml LPS, 3 μg/ml poly(I:C) + 5Z-7, 10 μg/ml poly(I:C) + 5Z-7, 10 ng/ml LPS + 5Z-7 for 5 h. **c** *Abin-1*^*+/+*^ MEFs and *Abin-1*^*−/−*^ MEFs were treated with poly(I:C), poly(I:C) + 5Z-7, poly(I:C) + 5Z-7+zVAD, LPS + 5Z-7, LPS + 5Z-7+zVAD for 5 h in presence or absence of Nec-1s. **d**, **e** *Tak1*^*+/+*^ MEFs and *Tak1*^−*/*−^ MEFs were transfected with negative control (NC) siRNA or *Abin-1* siRNA for 36 h followed by stimulation with poly(I:C), poly(I:C) + 5Z-7 (P5) for 6 h in presence or absence of Nec-1s. *Tak1*^*+/+*^ MEFs (**d**); *Tak1*^*−/−*^ MEFs (**e**). **f** *Abin-1*^*+/+*^ MEFs and *Abin-1*^*−/−*^ MEFs were treated with P5 or poly(I:C) + 5Z-7+zVAD (P5Z) for 4 h in presence or absence of Nec-1s, and cells were lysed for western blot analysis for apoptosis markers cleaved caspase-3, cleaved PARP-1, cleaved caspase-8, and cleaved CYLD. **g** *Abin-1*^*+/+*^*Ripk1*^*+/+*^, *Abin-1*^*−/−*^*Ripk1*^*+/+*^, *Abin-1*^*−/−*^*Ripk1*^*+/D138N*^, and *Abin-1*^−*/−*^*Ripk1*^*D138N/D138N*^ MEFs were treated with P5, P5Z for 5 h in the presence or absence of Nec-1s. D138N mutation on RIPK1 leads to RIPK1 kinase death. **h** *Abin-1*^*+/+*^ and *Abin-1*^−*/*−^ MEFs were treated with P5 for 4.5 h or 7.5 h in the presence or absence of Nec-1s, and then cells were subjected to immunoprecipitation with phospho-RIPK1 S166 antibody followed by western blot analysis of RIPK1. **i**–**k** *Abin-1*^*+/+*^ and *Abin-1*^*−/−*^ MEFs were treated with P5 for 4 h (**i**), 6 h (**j**), or 24 h (**k**) in the presence or absence of Nec-1s, RIPK3 inhibitor GSK-872, or Nec-1s+GSK-872. Supernatants were collected at indicated time points for ToxiLight assay. **l**, **m** *Abin-1*^*+/+*^ and *Abin-1*^−*/*−^ MEFs were transfected with NC siRNA, *Ripk3* siRNA, or *Mlkl* siRNA for 36 h and treated with P5 and P5Z for 5 h (**l**) or 7.5 h (**m**) in the presence or absence of Nec-1s. **n** *Abin-1*^*+/+*^ *Ripk3*^*+/+*^, *Abin-1*^−*/−*^*Ripk3*^*+/+*^, and *Abin-1*^*−/−*^*Ripk3*^−*/*−^ MEFs were treated with P5, P5Z for 7.5 h in the presence or absence of Nec-1s. **o** *Abin-1*^*+/+*^ MEFs and *Abin-1*^−*/*−^ MEFs were treated with P5 or P5Z for 5 h in the presence or absence of Nec-1s, and then cells were lysed for western blot analysis of p-MLKL, MLKL, cleaved PARP-1, and cleaved caspase-3. **p** *Abin-1*^*+/+*^ MEFs and *Abin-1*^−*/*−^ MEFs were treated with P5 for 0–16 h, and cells were lysed for western blot analysis of p-MLKL, MLKL, cleaved PARP-1, and cleaved caspase-3. **q** *Abin-1*^*−/−*^ MEFs were pre-incubated with 1 μg/ml or 4 μg/ml TNF blocking antibody (TNF Ab) for 45 min, and then cells were incubated with P5 or TNF + 5Z-7 (T5) for 6 h. **r** *Abin-1*^*+/+*^, *Abin-1*^*+/*−^, and *Abin-1*^*−/*−^ MEFs were pre-incubated with XIAP inhibitor SM-164 for 45 min, and then cells were incubated with P5 for 5 h. **s** *Abin-1*^*+/+*^, *Abin-1*^*+/−*^, and *Abin-1*^−*/−*^ MEFs were treated with poly(I:C) for 0 h and 4 h, and cells were subjected to RNA extraction and qPCR analysis of TLR3. **t** *Abin-1*^*+/+*^ and *Abin-1*^*−/*−^ MEFs were transfected with NC, *Tlr3, or Rig-I* siRNA for 36 h and then treated with P5 or P5Z for 6 h. Poly(I:C), 10 μg/ml (unless otherwise indicated); 5Z-7, 0.5 μM; Nec-1s, 10 μM; zVAD, 20 μM; and GSK-872, 3 μM. *P < 0.05, **P < 0.01, or ***P < 0.001.

Previously, Oshima et al. and Dziedzic et al. demonstrated that ABIN-1 deficiency sensitizes TNF + cycloheximide (TC)-induced apoptosis and TNF + cycloheximide+zVAD (TCZ)-induced necroptosis in MEFs^15,16^. In addition, ABIN-1 deficiency transforms TC-induced apoptosis into a mixed cell death of necroptosis and RIPK1-independent apoptosis^16^. In this study, we further investigated if ABIN-1 deficiency sensitizes poly(I:C)-induced cell death. As shown in Fig. 1a, poly(I:C) alone hardly induced cell death in wild-type MEFs, while poly(I:C) alone can induce some cell death in *Abin-1*^*−/−*^ MEFs at 7 h and 24 h, which was blocked by RIPK1 kinase inhibitor Nec-1s. Since TAK1 has been reported to suppress RIPK1 activation^31,32^ and TNF with TAK1 inhibitor can induce RDA^3^, we investigated the sensitization of TAK1 inhibitor 5Z-7 in poly(I:C)-induced cell death. Surprisingly, TAK1 inhibitor 5Z-7 greatly enhanced poly(I:C)-induced cell death in *Abin-1*^*−/−*^ MEFs, but not in *Abin-1*^*+/+*^ MEFs. However, 5Z-7+LPS did not induce significant cell death (Fig. 1b). We further found that both poly(I:C) + 5Z-7 (P5) and poly(I:C) + 5Z-7+zVAD (P5Z) can induce strong cell death in *Abin-1*^*−/−*^ MEFs, which can be blocked by Nec-1s (Fig. 1c). Moreover, poly(I:C) + cycloheximide (PC) or poly(I:C) + zVAD also induced moderate cell death in *Abin-1*^*−/−*^ MEFs, but not in *Abin-1*^*+/+*^ MEFs (Supplementary Fig. S1a, b). To exclude potential off-target effects of 5Z-7, we treated *Tak1*^*−/*−^ and *Tak1*^*+/+*^ MEFs with poly(I:C) alone or P5, and found that poly(I:C) alone induced a similar level of cell death as P5 in *Tak1*^*−/−*^ MEFs with ABIN-1 knocked down. In contrast, P5 induced more cell death than poly(I:C) alone in *Tak1*^*+/+*^ cells with ABIN-1 knocked down (Fig. 1d, e and Supplementary Fig. S1c). We further observed that P5 induced dramatic caspase-3, caspase-8, and PARP-1 cleavage, which was blocked Nec-1s, indicating that P5 may induce RIPK1 kinase-dependent apoptosis in *Abin-1*^*−/−*^ cells. As a control, P5Z did not activate these apoptosis markers (Fig. 1f). Consistently, RIPK1 kinase-dead knockin mutation D138N blocked P5 or P5Z-induced cell death in *Abin-1*^*−/−*^ MEFs in a dose-dependent manner (Fig. 1g). Western blot analysis showed that P5 can induce RIPK1 S166 phosphorylation in *Abin-1*^*−/−*^ MEFs (Fig. 1h).

Interestingly, we found that P5-induced cell death can be completely inhibited by RIPK3 inhibitor GSK-872 at an earlier time point (4 h) (Fig. 1i), and partially inhibited by GSK-872 at a later time point (6 h) (Fig. 1j), and eventually uninhibited by GSK-872 in *Abin-1*^−*/*−^ MEFs (Fig. 1k). However, P5-induced cell death can be completely inhibited by Nec-1s at all the time points (4 h, 6 h, and 24 h) (Fig. 1i–k). This implies that cells could die in a necroptotic way in the early stage upon P5 stimulation in *Abin-1*^−*/*−^ MEFs, while cells mainly die in RDA in the late stage. This finding was further validated using a RIPK1/MLKL RNAi system. We found that P5-induced cell death was completely inhibited by RIPK3 or MLKL knockdown in *Abin-1*^*−/−*^ MEFs at an early time (5 h), whereas partial inhibition by RIPK3 or MLKL knockdown occurred at a later time (7.5 h) (Fig. 1l, m and Supplementary Fig. S1d, e). Deletion of *Ripk3* also partially suppressed P5-induced cell death in *Abin-1*^*−/−*^ MEFs (Fig. 1n). As a control, P5Z-induced cell death was completely inhibited by RIPK3/MLKL knockdown or *Ripk3* knockout (Fig. 1l–n). Western blot analysis further revealed that P5 induced MLKL phosphorylation and caspase-3/PARP-1 cleavage simultaneously (Fig. 1o, p), which are recognized as the critical markers for necroptosis activation and apoptosis activation, respectively. In contrast, P5Z induced p-MLKL but not caspase-3/PARP-1 cleavage, indicating that P5Z only induces necroptosis, while P5 induces both RDA and necroptosis in ABIN-1 deficient MEFs (Fig. 1o, p).

To exclude the possibility that P5-induced cell death is because of a secondary effect of poly(I:C)-induced TNF, we pre-treated cells with TNF neutralization antibody and stimulated cells with P5 or TNF + 5Z-7 (T5). It was shown that P5-induced cell death in *Abin-1*^*−/−*^ MEFs was not blocked by TNF antibody, while T5-induced cell death was dramatically attenuated by TNF antibody in a dose-dependent manner (Fig. 1q). This proves that P5-induced cell death is irrelevant to potential secondary effect of TNF. In fact, we were unable to detect any TNF secretion in *Abin-1*^*−/−*^ MEFs in response to poly(I:C) stimulation. Moreover, ubiquitin ligase IAP antagonists SM-164 did not sensitize P5-induced cell death in *Abin-1*^*−/−*^ MEFs (Fig.1r), which implies that P5-induced cell death may not involve ubiquitination regulation. To investigate the potential mechanisms by which ABIN-1 deficiency boosts P5- and P5Z-induced cell death, we examined TLR3 expression level because engagement of TLR3 can trigger TRIF-mediated cell death via apoptosis or necroptosis. As showed in Fig.1s, ABIN-1 deficiency not only enhanced the basal expression of TLR3 but also enhanced poly(I:C)-induced TLR3 expression. We further demonstrated that TLR3, but not Rig-I or MDA5, was the critical mediator for P5- or P5Z-induced cell death in *Abin-1*^*−/−*^ MEFs by RNA interference experiment (Fig. 1t and Supplementary Fig. S1f–i). Taken together, P5Z only induces necroptosis, while P5 induces both RDA and necroptosis concurrently in ABIN-1-deficient MEFs. Upon P5 stimulation, cells die by necroptosis at the early stage and die by RDA in the late stage. The higher basal and poly(I:C)-induced level of TLR3 in *Abin-1*^*−/*−^ MEFs could partially explain the stronger cell death induced by poly(I:C)/P5/P5Z in *Abin-1*^*−/*−^ MEFs.

### ABIN-1 deficiency sensitizes colorectal cancer cells to P5-induced RIPK1 kinase-independent apoptosis and poly(I:C) + 5Z-7-oxozeaenol + IDN-6556 (P5I)-induced necroptosis

**Fig. 2:**
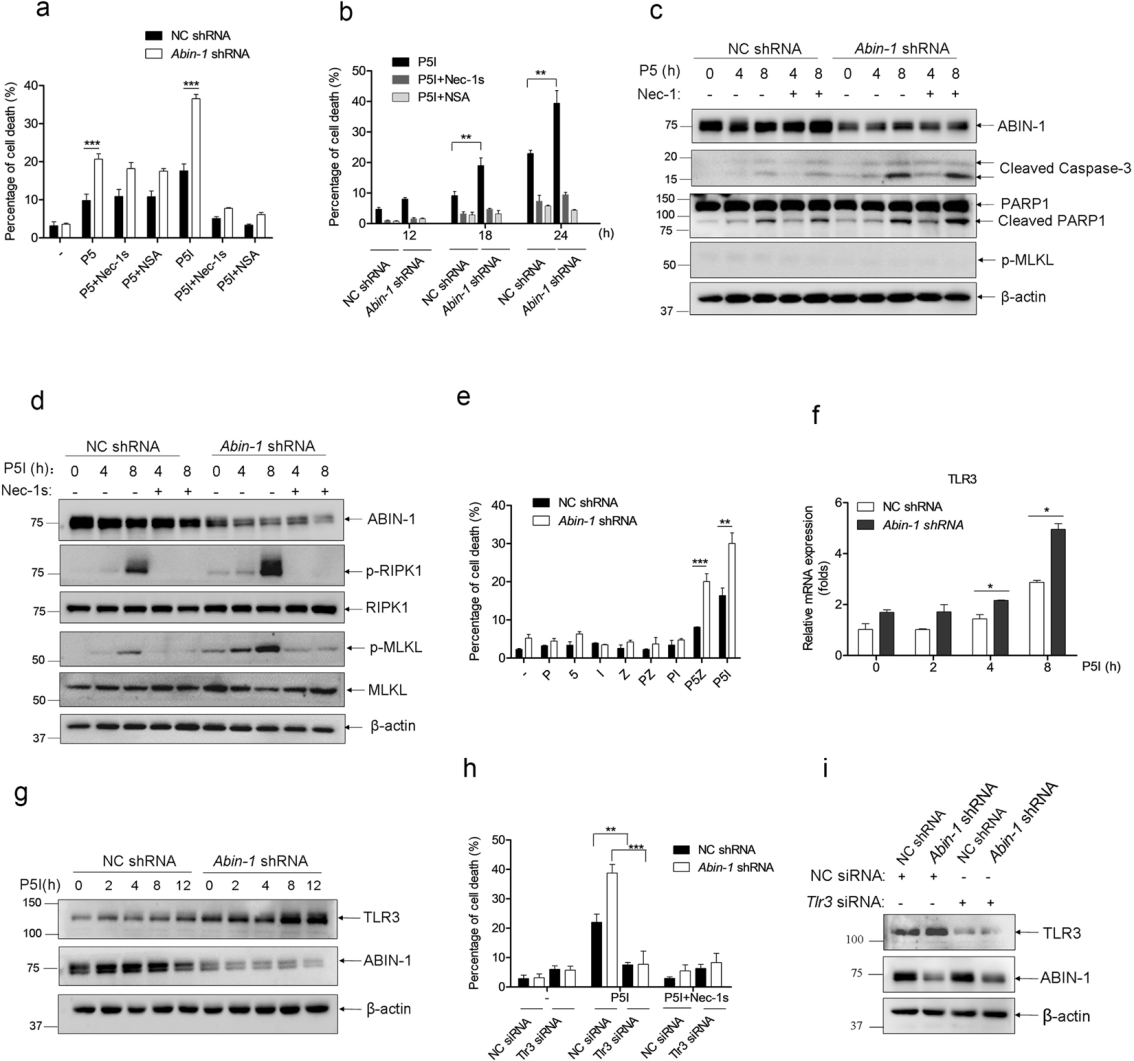
ABIN-1 deficiency sensitizes colorectal cancer cells to poly(I:C)-induced cell death. **a** COLO205 cells were transduced with lentivirus-based control shRNA or *Abin-1* shRNA and screened with puromycin for 5 days. Then, cells were treated with poly(I:C) + 5Z-7-oxozeaenol (P5) or poly(I:C) + 5Z-7-oxozeaenol+IDN-6556 (P5I) with or without Nec-1s or necrosulfonamide (NSA) for 24 h, and cell deaths were measured by ToxiLight assay. **b** Time course of P5I-induced cell death. NC shRNA and *Abin-1* shRNA COLO205 cells were treated with P5I for 12, 18, or 24 h with or without Nec-1s or NSA. **c** COLO205 NC shRNA and *Abin-1* shRNA cells were treated with P5 for 4 h or 8 h with or without Nec-1s, and then cells were lysed at indicated time points, followed by western blot analysis of ABIN-1 and apoptosis markers cleaved caspase-3 and PARP-1 and necroptosis marker p-MLKL. **d** COLO205 NC shRNA and *Abin-1* shRNA cells were treated with P5I for 4 h or 8 h with or without Nec-1s, and then cells were lysed at indicated time points, followed by western blot analysis of ABIN-1, RIPK1, MLKL, and necroptosis markers p-RIPK1 and p-MLKL. **e** Comparison of poly(I:C) (P)-, 5Z-7-oxozeaenol^5^-, IDN-6556 (I)-, zVAD (Z)-, poly(I:C) + zVAD (PZ)-, poly(I:C) + IDN-6556 (PI)-, poly(I:C) + 5Z-7+zVAD (P5Z)-, and P5I-induced cell deaths. Drug treatments for 24 h. **f** COLO205 NC shRNA and *Abin-1* shRNA cells were treated with P5I for 0–8 h, and the total RNA was extracted and subjected to the qPCR assay of TLR3 expression. **g** COLO205 NC shRNA and *Abin-1* shRNA cells were treated with P5I for 0–12 h, and cells were lysed and subjected to western blot analysis of ABIN-1 and TLR3. **h**, **i** COLO205 NC shRNA and *Abin-1* shRNA cells were transfected with NC siRNA or *Tlr3* siRNA and incubated for 24 h, followed by P5I treatment for another 24 h in the presence or absence of Nec-1s. Cell deaths were detected by ToxiLight assay (**h**), and knockdown efficiency of TLR3 and ABIN-1 were analyzed by western blot (**i**). Poly(I:C), 10 μg/ml; 5Z-7, 0.5 μM; IDN-6556, 2.5 μM; Nec-1s, 10 μM; zVAD, 20 μM; and NSA, 5 μM. *P < 0.05, **P < 0.01, or ***P < 0.001.

Next, we investigated the effect of ABIN-1 deficiency on poly(I:C)-induced necroptosis in CRC cells. Here, we used IDN-6556 to substitute the zVAD, as IDN-6556 is a clinically-used caspases inhibitor with the same effect of zVAD, but with lower toxicity. In contrast to MEFs, P5 and P5I can induce cell death either in wild-type or in ABIN-1-deficient CRC cells. ABIN-1 deficiency sensitized both P5- and P5I-induced cell deaths (Fig. 2a, b). Unlike MEFs, P5-induced cell death cannot be inhibited by Nec-1s and MLKL inhibitor NSA, but P5I-induced cell death can be completely inhibited by Nec-1s and NSA in COLO205 cells (Fig. 2a, b). To prove the TAK1 specificity, we treated control siRNA-transfected and *Tak1* *siRNA*-transfected COLO205 cells with poly(I:C) alone or P5, and found that poly(I:C) + 5Z-7 induced more cell death than poly(I:C) alone in control siRNA-transfected cells, while poly(I:C) alone and P5 induced a similar degree of cell death in TAK1 knockdown cells (Supplementary Fig. S2a, b). Western blot analysis showed that P5 induced caspase-3 and PARP-1 cleavage, but not MLKL phosphorylation in CRC cells and ABIN-1 deficiency enhanced the caspase-3/PARP-1 cleavage, which was not inhibited by Nec-1s (Fig. 2c and Supplementary Fig. S2c). P5I induced RIPK1 S166 phosphorylation and MLKL phosphorylation in CRC cells, which was amplified by ABIN-1 deficiency (Fig. 2d and Supplementary Fig. S2c), which was blocked by Nec-1s. These data suggest that P5-induced cell death in CRC cells is RIPK1 kinase-independent apoptosis and P5I-induced cell death is necroptosis. We further observed that poly(I:C) alone, 5Z-7-oxozeaenol alone, IDN-6556 alone, zVAD alone, poly(I:C) + IDN-6556 (PI), or poly(I:C) + zVAD (PZ) did not induce significant cell death in COLO205 cells, while P5I or P5Z induced dramatic cell death. Knocking down ABIN-1 enhanced both P5Z- and P5I-induced necroptosis (Fig. 2e). We subsequently explored the mechanism underlying the enhanced sensitivity of poly(I:C)-induced cell death in ABIN-1-knocked down CRC cells. In consistent to MEFs, ABIN-1 knockdown in COLO205 cells markedly increased P5I-induced TLR3 mRNA and protein levels (Fig. 2f, g), and TLR3 knockdown eliminated P5I-induced cell death in both ABIN-1-knockdown and its control cells confirming the importance of TLR3 in P5I-induced necroptotic cell death (Fig. 2h, i). Since ABIN-1 deficiency has been shown to enhance inflammation and innate immune responses^17^, it is most likely that increased production of inflammatory cytokines in ABIN-1-deficient cells leads to increased TLR3 expression.

### ABIN-1 deficiency sensitizes colorectal cancer cells to TNF + birinapant + zVAD/IDN-6656- and TNF + 5-fluorouracil + zVAD/IDN-6556-induced necroptosis

**Fig. 3:**
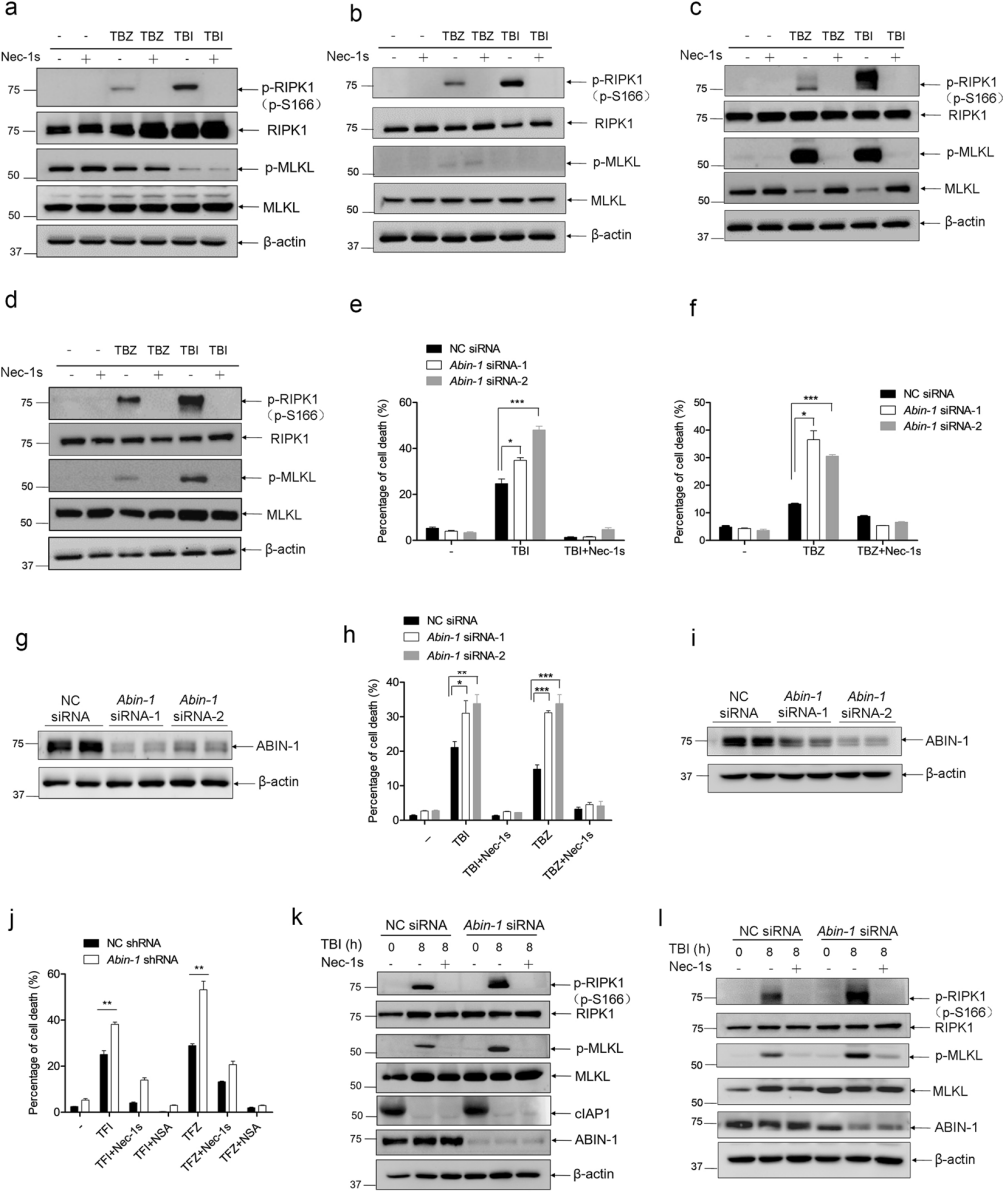
ABIN-1 deficiency sensitizes colorectal cancer cells to TNF + birinapant + zVAD/IDN-6656- and TNF + 5-fluorouracil + zVAD/IDN-6556-induced necroptosis. **a**–**d** Four different human CRC cell lines were treated with TNF + birinapant+zVAD (TBZ) or TNFα + birinapant+IDN-6556 (TBI) for 8 h in the presence or absence of Nec-1s. Cells were lysed by RIPA buffer at 8 h and necroptosis markers phospho-RIPK1 (p-RIPK1), RIPK1, phospho-MLKL (p-MLKL), and MLKL were detected by western blot. TNF, 10 ng/ml; birinapant, 100 nM; zVAD, 20 μM; Nec-1s, 10 μM; and IDN-6556, 2.5 μM. Caco-2, human colorectal adenocarcinoma cell (**a**); HCT116, human colorectal carcinoma cell line (**b**); HT-29, human colorectal adenocarcinoma cells (**c**); and COLO205, human colorectal adenocarcinoma cells (**d**). **e**–**g** HT-29 cells were transfected with two different *Abin-1* siRNAs and incubated for 24 h. Then, cells were re-plated and treated with TNF + birinapant + IDN-6556 (TBI) or TNF + birinapant+zVAD (TBZ) for 8 h in the presence or absence of Nec-1s. Cell deaths were measured by ToxiLight assay. TBI treatments + /− Nec-1s (**e**); TBZ treatments + /− Nec-1s (**f**); and Knockdown efficiency in HT-29 cells (**g**). Proteins were extracted from HT-29 cells 32 h after siRNAs transfection. Each siRNA sample was loaded in duplicates. **h**–**i** COLO205 cells were transfected with two different *Abin-1* siRNAs and incubated for 24 h. Then, cells were re-plated and treated with TBI or TBZ for 8 h in the presence or absence of Nec-1s. Cell deaths were measured by ToxiLight assay (**h**); knockdown efficiency (**i**). **j** HT-29 cells were transduced with lentivirus-based control shRNA or *Abin-1* shRNA and screened with puromycin for 5 days. Then, cells were treated with TNF + 5-fluorouracil+IDN-6556 (TFI) or TNF + 5-fluorouracil+zVAD (TFZ) for 36 h in the presence or absence of Nec-1s or necrosulfonamide (NSA). **k**, **l** HT-29 cells or COLO205 cells were transfected with control siRNA or *Abin-1* siRNA and incubated for 24 h. Then, cells were re-plated and treated with TBI for 8 h in the presence or absence of Nec-1s. Cell lyses were subjected to western blot and p-RIPK1, RIPK1, p-MLKL, MLKL, cIAP1, and ABIN-1 were detected. Cell deaths were measured by ToxiLight assay. HT-29 cells TBI treatments + /− Nec-1s **(k)**; and COLO205 cells TBI treatments + /− Nec-1s (**l**). TNFα, 10 ng/ml; birinapant, 100 nM; zVAD, 20 μM; Nec-1s, 10 μM; IDN-6556, 2.5 μM; NSA, 5 μM; and 5-FU, 250 μM. *P < 0.05, **P < 0.01, or ***P < 0.001.

Next, we explored if ABIN-1 deficiency contributes to TNF-triggered necroptosis in CRC cells. It is known that >80% of cancer cell lines are resistant to necroptosis because of incomplete necroptotic machinery, and RIPK3 and MLKL deficiencies are commonly found in numerous cancer cell lines^22,33^. Therefore, we first screened for CRC cell lines that are sensitive to TNF-triggered necroptosis. We chose four common CRC cell lines, Caco-2, HCT116, HT-29, and COLO205, and treated those cells with necroptosis inducers TNF + birinapant+zVAD (TBZ) or TNF + birinapant+IDN-6556 (TBI) in the presence or absence of necrostatin-1 (Nec-1s). We observed that TBZ or TBI can induce RIPK1 phosphorylation in all of those four cells, which was blocked by RIPK1 kinase inhibitor Nec-1s (Fig. 3a–d); however, TBZ or TBI only induced MLKL phosphorylation in HT-29 and COLO205 cells but not in Caco-2 and HCT116 cells (Fig. 3a–d), indicating Caco-2 and HCT116 are defective in the necroptotic signaling pathway and inappropriate for the necroptosis study. Thus, we mainly focused on HT-29 and COLO205 cells in the following study.

Using RNA interference, we found that knockdown of ABIN-1 in HT-29 cells by two different siRNA oligos both increased TBI- or TBZ-induced cell death (Fig. 3e–g), which was blocked by Nec-1s. Similar results were observed in COLO205 cells (Fig. 3h, i). Consistently, transient overexpression of ABIN-1 in HT-29 reduced TBI-induced RIPK1-dependent necroptosis (Supplementary Fig. S3a, b). In addition, knockdown of ABIN-1 sensitized HT-29 cells to TNF + 5-fluorouracil+IDN-6556 (TFI)- or TNF + 5-fluorouracil+zVAD (TFZ)-induced cell death, which was blocked by Nec-1s and MLKL inhibitor necrosulfonamide (NSA) (Fig. 3j). To further examine the sensitization effect of ABIN-1 deficiency on necroptosis, we analyzed two necroptosis markers, phospho-RIPK1, and phospho-MLKL. As expected, knockdown of ABIN-1 in HT-29 or COLO205 cells significantly increased TBI-induced phosphorylation of RIPK1 and MLKL, which can be blocked by Nec-1s (Fig. 3k, l). Taken together, these results prove that ABIN-1 deficiency sensitizes CRC cells to TNF-triggered necroptosis.

### ABIN-1 deficiency augments necroptosis in colorectal cancer cells triggered by birinapant + IDN-6556/zVAD or 5-fluorouracil + IDN-6556/zVAD

**Fig. 4:**
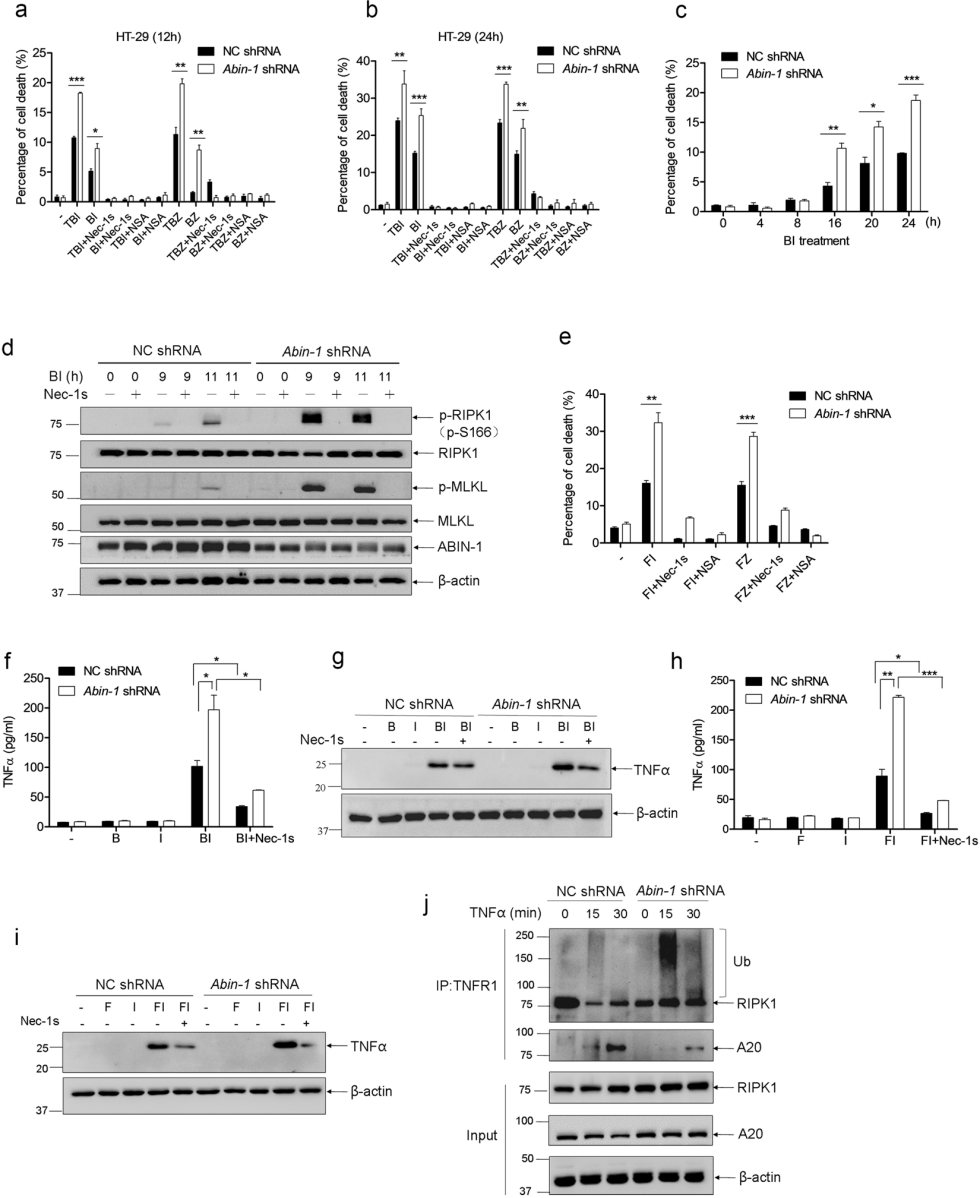
ABIN-1 deficiency augments necroptosis in colorectal cancer cells triggered by birinapant + IDN-6556/zVAD or 5-fluorouracil + IDN-6556/zVAD. **a, b** HT-29 cells were transduced with lentivirus-based control shRNA (NC shRNA) or *Abin-1* shRNA and screened with puromycin for 5 days. Then, cells were treated with TNFα + birinapant+IDN-6556 (TBI), birinapant+IDN-6556 (BI), TNFα + birinapant+zVAD (TBZ), or birinapant+zVAD (BZ) for 12 h or 24 h in the presence or absence of Nec-1s or NSA. Drug treatments for 12 h (**a**); Drug treatments for 24 h (**b**); **c** HT-29 NC shRNA and *Abin-1* shRNA cells were treated with BI for 0, 4, 8, 16, 20, or 24 h, and cell deaths were measured by ToxiLight assay. **d** HT-29 NC shRNA and *Abin-1* shRNA cells were treated with BI for 9 h or 11 h in the presence or absence of Nec-1s. Cell lysates were detected with p-RIPK1, RIPK1, p-MLKL, MLKL, and ABIN-1 by western blot. **e** HT-29 NC shRNA and *Abin-1* shRNA cells were treated with 5-fluorouracil+IDN-6556 (FI) or 5-fluorouracil+zVAD (FZ) for 36 h in the presence or absence of Nec-1s or NSA. **f**, **g** HT-29 NC shRNA and *Abin-1* shRNA cells were treated with birinapant (B), IDN-6556 (I), BI, or BI + Nec-1s for 12 h, and supernatants were collected for ELISA analysis of TNF (**f**), and cells were lysed for western blot analysis of TNF (**g**). **h**, **i** HT-29 NC shRNA and *Abin-1* shRNA cells were treated with 5-fluorouracil (F), I, FI, or FI + Nec-1s for 36 h, and supernatants were collected for ELISA analysis of TNF (**h**) and cells were lysed for western blot analysis of TNF (**i**). **j** HT-29 NC shRNA and *Abin-1* shRNA cells were treated with TNF for 0, 15, or 30 min and then immunoprecipitated with TNFR1 antibody, followed by western blot analysis for RIPK1 and A20. TNF, 10 ng/ml for cell death and 50 ng/ml for IP; birinapant, 100 nM; 5-FU, 250 μM; zVAD, 20 μM; Nec-1s, 10 μM; IDN-6556, 2.5 μM; and NSA, 5 μM. *P < 0.05, **P < 0.01, or ***P < 0.001.

Considering TNF is a strong innate immune stimulus and may lead to endotoxin shock if given a high dose, a drug combination containing TNF may be a restriction in future clinical application. Therefore, we wondered if birinapant + IDN-6556 (BI) or birinapant + zVAD (BZ) can trigger necroptosis in CRC cells in absence of TNF. As shown in Fig. 4a and b, BI- and BZ-induced cell death was detectable at 12 h and markedly increased at 24 h to a level comparable to that induced by TBI or TBZ, which was blocked by necroptosis inhibitors Nec-1s and NSA. Notably, knockdown of ABIN-1 sensitized TBI-, TBZ-, BI-, or BZ-induced necroptosis. A time-course experiment showed that ABIN-1 deficiency strengthened BI-induced cell death at multiple time points (Fig. 4c). Consistently, ABIN-1 deficiency dramatically enhanced the necroptosis markers phospho-RIPK1 and phospho-MLKL upon BI treatment (Fig. 4d and Supplementary Fig. S4). Not only restricted to BI or BZ, 5-fluorouracil+zVAD (FZ) or 5-fluorouracil+IDN-6556 (FI) also induced strong necroptosis in HT-29 cells, and ABIN-1 deficiency further amplified the FZ- or FI -induced necroptosis (Fig. 4e).

To further explore the reason that BI induces necroptosis in absence of TNF, we investigated whether these chemotherapy drugs induce TNF secretion in CRC cells.

Interestingly, although birinapant alone or IDN-6556 alone did not yield increased levels of intracellular TNF and TNF secretions, a combination of birinapant and IDN-6556 resulted in a considerable increase in levels of intracellular TNF and TNF secretions. ABIN-1 deficiency doubled BI-induced TNF expression and secretion (Fig. 4f, g). Suppression of RIPK1 kinase activity by Nec-1s decreased BI-induced TNFα secretion by 70% (Fig. 4f). Not restricted to BI, FI also induced TNF secretion in CRC cells, and ABIN-1 deficiency further enhanced intracellular and extracellular TNF levels, which can be inhibited by Nec-1s (Fig. 4h, i). These results suggest that increased TNF in ABIN-1-deficient CRC cells may explain the higher sensitivity of ABIN-1 deficient CRC cells to BI- or FI-induced necroptosis compared to wild-type.

### ABIN-1 deficiency augments TNF-induced necroptosis by regulating A20-mediated RIPK1 deubiquitination

We further investigated other mechanisms by which ABIN-1 deficiency sensitizes CRC cells to TNFα-related necroptosis. Previously, we have revealed that in normal cells, ABIN-1 deficiency promotes RIPK1 K63 polyubiquitination and activation of RIPK1 by reducing the recruitment of ubiquitin-editing enzyme A20 to TNF-RSC upon TNF stimulation^16^. We investigated this mechanism in CRC cells that possess a highly variable genome. We found that RIPK1 ubiquitination in TNF-RSC was greatly increased in ABIN-1-deficient HT-29 cells upon TNFα stimulation. A20 was less recruited to TNF-RSC in ABIN-1-deficient cells compared to control cells (Fig. 4j). These results suggest that CRC cells and normal cells share the same mechanism of necroptosis stimulation.

### ABIN-1 deficiency enhances necroptosis of human colorectal cancer xenograft

**Fig. 5:**
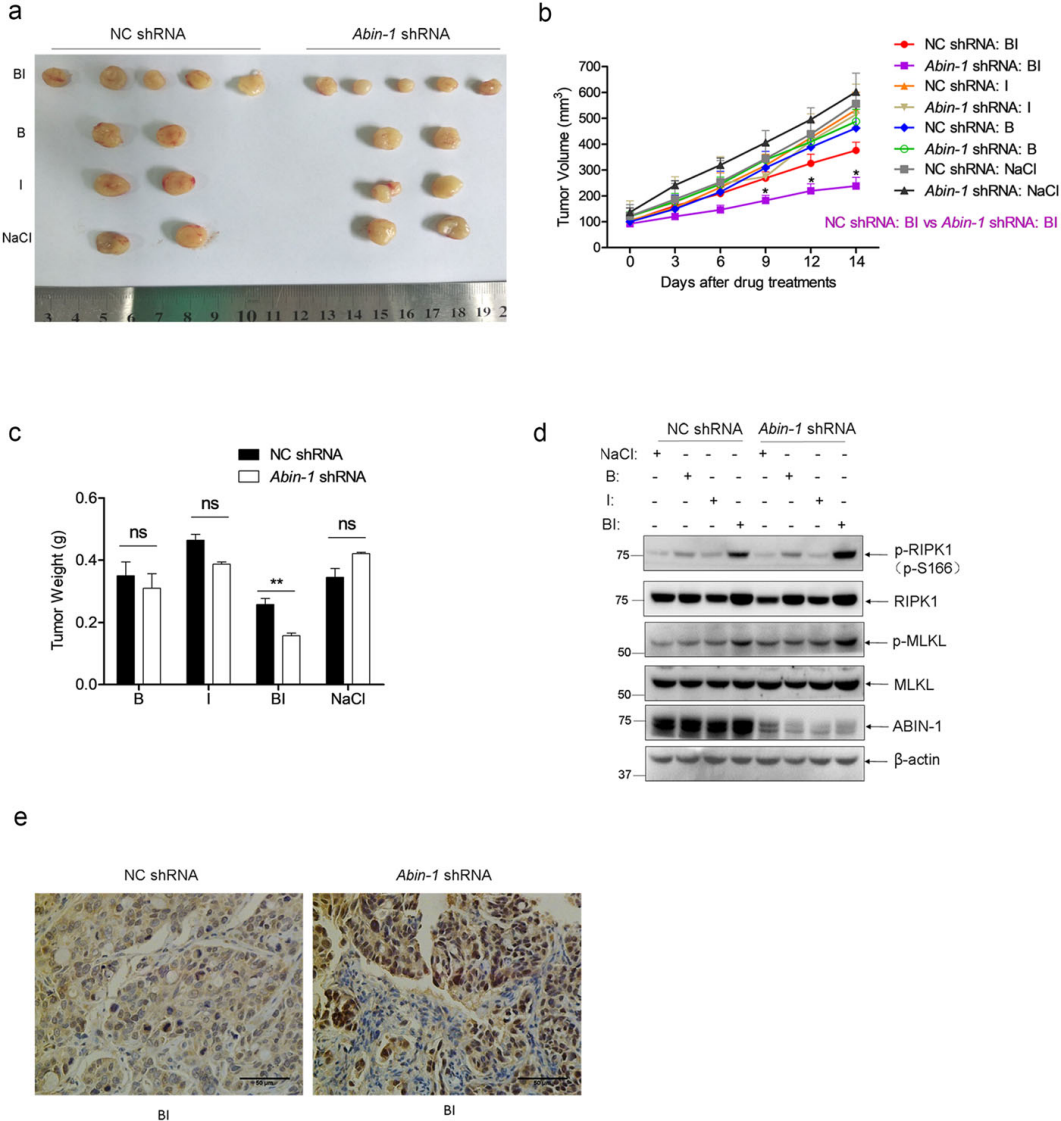
ABIN-1 deficiency improves birinapant + IDN-6556-induced necroptosis cancer therapy. HT-29 cells were transduced with lentivirus-based control shRNA (NC shRNA) or *Abin-1* shRNA and screened with puromycin for 5 days to establish stable knockdown pool cells. NC shRNA cells and *Abin-1* shRNA cells were subcutaneously injected into the left and right flank of BALB/c nude mice, respectively. When tumors grew to 100 mm^3^, mice were divided into four groups (normal saline (NaCl), *n* = 2; IDN-6556 (I), *n* = 2; birinapant (B), *n* = 2; birinapant + IDN-6556 (BI), *n* = 5). Mice were intraperitoneally injected with indicated drugs every three days. Tumor volumes were measured every 2 or 3 days, and mice were sacrificed 14 days after the first drug injection. Tumors were isolated for imaging, weighing, western blot, and immunohistochemical staining. Birinapant, 2.5 mg/kg and IDN-6556, 1.25 mg/kg. **a** Tumors images. **b** Tumor volumes. Volume (mm^3^) = 0.5 × length (mm) × width^2^ (mm^2^). **c** Tumor weights (g). **d** Western blot analysis for necroptosis markers. Each lane was loaded with a randomly selected tumor sample from each group. **e** Immunohistochemical staining with phospho-RIPK1 of tumor samples in BI group. *P < 0.05, **P < 0.01, or ***P < 0.001.

To further investigate if ABIN-1 deficiency improves necroptosis-based cancer therapy, we established a xenograft model by injecting control shRNA CRC cells and *Abin-1* shRNA CRC cells into the left flank and right flank of nude mice, respectively, to evaluate if *Abin-1* deficiency could enhance necroptosis of tumor cells in vivo. First, we investigated whether ABIN-1 deficiency improves TNF-induced necroptosis in the HT-29 xenograft model. As shown in Fig. 5a (tumor images) and 5b (tumor volume), BI treatment partially inhibited tumor growth compared with normal saline treatment; however, birinapant alone or IDN-6556 alone had no effect. Notably, ABIN-1 deficiency significantly enhanced BI-induced tumor suppression as evidenced by tumor image, tumor volume (*P* < 0.05 at days 9, 12, and 14), and tumor weight (Fig. 5a–c). To verify that the BI-induced tumor suppression was from necroptosis, western blot analysis of necroptosis markers phospho-RIPK1 and phospho-MLKL in tumor samples was performed. Western blot results showed that BI induced moderate upregulation of RIPK1 and MLKL phosphorylation in control tumors; however, ABIN-1 knockdown greatly increased BI-induced RIPK1 and MLKL phosphorylation (Fig. 5d and Supplementary Fig. S5). Immunohistochemical staining results also supported that ABIN-1 deficiency enhanced RIPK1 phosphorylation in response to BI treatment (Fig. 5e).

**Fig. 6:**
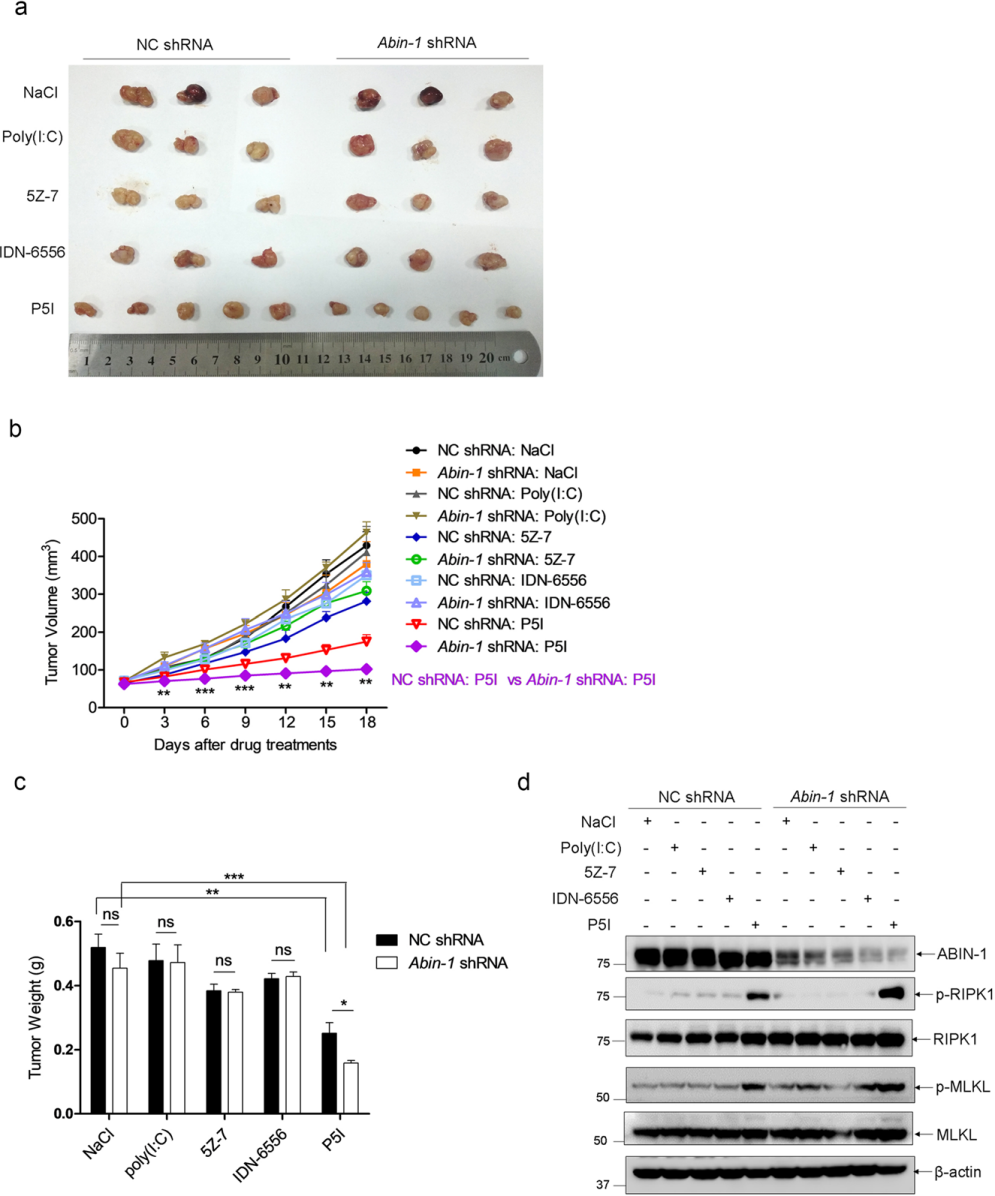
ABIN-1 deficiency improves poly(I:C) + 5Z-7-oxozeaenol + IDN-6556-induced necroptosis cancer therapy. COLO205 cells were transduced with lentivirus-based control shRNA (NC shRNA) or *Abin-1* shRNA and screened with puromycin for 5 days to establish stable knockdown pool cells. NC shRNA cells and *Abin-1* shRNA cells were subcutaneously injected into the left flank and right flank of BALB/c nude mice, respectively. When tumors grew to 100 mm^3^, mice were divided into five groups (normal saline (NaCl), *n* = 3; poly(I:C), *n* = 3; 5Z-7-oxozeaenol (5Z-7), *n* = 3; IDN-6556, *n* = 3; poly(I:C) + 5Z-7-oxozeaenol+IDN-6556 (P5I), *n* = 5), Mice were intraperitoneally injected with indicated drugs every 3 days. Tumor volumes were measured every three days, and mice were sacrificed 18 days after the first drug injection. Tumors were isolated for imaging, weighing, and western blot analysis. Poly(I:C), 5 mg/kg; IDN-6556, 1.25 mg/kg; and 5Z-7-oxozeaenol, 0.6 mg/kg. **a** Tumors images. **b** Tumor volumes. Volume (mm^3^) = 0.5 × length (mm) × width^2^ (mm^2^). **c** Tumor weights (g). **d** Western blot analysis for necroptosis markers. Each lane was loaded with a randomly selected tumor sample from each group. *P < 0.05, **P < 0.01, or ***P < 0.001.

Furthermore, we explored if ABIN-1 deficiency improves poly(I:C)-induced necroptotic therapy in the COLO205 xenograft model. As shown in Fig. 6a–c, 4 mg/kg poly(I:C) or 1.25 mg/kg IDN-6556 did not significantly inhibit tumor growth, and 0.6 mg/kg 5Z-7-oxozeaenol only minimally inhibited tumor growth, but P5I induced robust tumor suppression by 50–60%. ABIN-1 deficiency further enhanced P5I-induced tumor suppression flattened the tumor growth curve and reduced the tumor volume and weight (Fig. 6a–c). Western blot analysis of phospho-RIPK1 and phospho-MLKL also supported that active necroptosis occurred in P5I-treated tumors, and ABIN-1 deficiency further enhanced the necroptosis (Fig. 6d). Taken together, we conclude that suppression of ABIN-1 enhances the therapeutic effect of necroptosis-based cancer therapy and increases the probability of slowing tumor progression.

## Discussion

In this study, we found that ABIN-1 is a key suppressor for poly(I:C)-induced RDA and necroptosis in MEFs. In the presence of ABIN-1, P5 is insufficient to induce RDA or necroptosis, while deficiency of ABIN-1 permits cells to undergo RDA and necroptosis. Upon poly(I:C) stimulation, TLR3 is engaged and programmed to recruit a complex composed of TRIF, cIAPs, caspase-8, FADD, and RIPK1 to trigger cell death. ABIN-1 deficiency dramatically increases TLR3 expression and may help to recruit more active RIPK1 to initiate RDA and necroptosis. Usually, apoptosis and necroptosis cannot occur at the same time because caspase-8 can cleave RIPK1 at site Asp325 and inhibit the RIPK1 kinase activation and subsequent necroptosis. The classic RDA inducer TNF + 5Z-7-oxozeaenol cannot induce RDA and necroptosis simultaneously^3^. However, ABIN-1 deficiency breaks through the barrier between apoptosis and necroptosis. Previously, we observed that a classic apoptosis inducer TC can induce RIPK1-independent apoptosis and necroptosis concurrently in *Abin-1*^−*/−*^ MEFs. Here, we further reveal that P5 can induce RDA and necroptosis concurrently in *Abin-1*^*−/−*^ MEFs. Interestingly, P5 induces both caspases activation and MLKL phosphorylation in the early stage, but cells die predominantly in a necroptotic pathway; however, in the late stage, caspases-mediated RDA gradually transforms into the principal form of cell death. A possible explanation is ABIN-1 deficiency may dramatically elevate the RIPK1 kinase activity and promptly recruit RIPK3/MLKL to initiate necroptosis; In the meantime, active RIPK1 can recruit FADD, caspase-8 to mediate RDA, and active capase-8, in turn, cleaves RIPK1 to terminate necroptosis, but this process is slower than necrosome formation. Therefore, we first observed P5-induced necroptosis followed by mixed cell death of RDA and necroptosis and lastly only RDA. Contradictory to the results in MEFs, P5 induces RIPK1 kinase-independent apoptosis in CRC cells, but not a mixed cell death of RDA and necroptosis. Many factors have been reported to play a role in the regulation of RDA and necroptosis, such as NEK1, LRRK2, APC11, and c-Cbl for RDA and ZBP1, Parkin, MYC and A20 for necroptosis^3,19,34–36^. Therefore, it is possible that a cell type-specific regulator may block P5-induced RDA and necroptosis in ABIN-1-deficient CRC cells.

Necroptosis-based cancer therapy is a promising strategy to conquer chemotherapeutic drug resistance; however, not every cancer is suitable for this strategy. Najafov et al. report that 83% of cancer types do not undergo necroptosis. Specifically, in a screen of 941 cell lines, 780 are resistant to TNF + SM-164+zVAD (TSZ)-induced necroptosis. The loss of RIPK3 expression is the major contributing factor in the escape from necroptosis^33^. However, leukemia and CRC seem to be more sensitive to necroptosis compared with other cancer types, and ~50% of leukemia and 35% of intestine cancer cell lines are sensitive to TSZ-induced necroptosis^33^. Therefore, CRC is an appropriate cancer type for necroptosis-based therapy. Metzig et al. reported that 5-FU plus IDN-7314, a caspase inhibitor, achieved a better tumor-suppressive effect compared with 5-FU alone in an HT-29 xenograft model. Using 13 unique CRC patient samples, they found that >50% of CRC samples were more sensitive to pro-necroptosis agent 5-FU + zVAD than to pro-apoptosis agent 5-FU^27^. These findings suggest a promising benefit of necroptosis-based cancer therapy in CRC patients.

TNF + cIAPs inhibitors + caspase inhibitors is commonly used to induce necroptosis in vitro. However, this combination may not be applicable in vivo because high dose of TNF may evoke a strong inflammation leading to sepsis and multiple organ dysfunction^37^. In addition, since normal tissue may also respond to necroptosis inducers, more research needs to explore targeted necroptosis-based cancer therapy. Our study is one of a few to show FI and BI can induce TNF auto-secretion in cancer cells. Future clinical implications of our study are the possibility of reducing the number of cancer drugs in a regimen from three to two and offering targeted therapy to specific tumor sites since they have higher TNF levels compared to normal tissue. Notably, the combination of BI may be a promising necroptosis-based cancer therapy regimen because both birinapant, a phase I/II anti-cancer reagent and IDN-6556, an FDA-approved pan-caspase inhibitor have been well-studied for individual efficacy and safety profile, and the clinical use of these together may be warranted. In our xenograft experiment, BI treatment did not result in dramatic bodyweight loss or other side effects suggesting a low toxicity profile in BI-based necroptosis therapy in vivo.

Similar to BI, the pro-necroptosis combination P5I also had a robust necroptosis response and low toxicity in the xenograft model. Poly(I:C) is used in conjunction with chemotherapeutic drugs (e.g., cycloheximide, cIAP inhibitors, and arsenic trioxide) to induce apoptosis and play antitumor effects^38,39^. Poly(I:C) also serves as a cancer vaccine adjuvant mainly via facilitating dendritic cell maturation^40^. Takemura et al.^28^ reported that poly(I:C) + zVAD (PZ) can induce necroptosis in CT26 colorectal cells without secondary effects of TNFα or type I IFNs. However, our in vitro data showed that PZ and PI do not induce significant cell death in COLO205 cells. In contrast, P5Z and P5I induced more robust necroptosis (Fig. 2e). E6201, a related analog of 5Z-7-oxozeaenol, is in phase I/II clinical trials showing a good tolerance in the treatment of leukemia and solid tumors^41^. Therefore, we propose that poly(I:C) + E6201 + IDN-6556 may be a promising necroptosis inducer in cancer therapy.

For clinical application in necroptosis-based cancer therapy, there are three potential strategies to suppress ABIN-1: (1) develop small molecule inhibitors to block the interaction of ABIN-1 and A20, as this interaction is important for the recruitment of A20 to TNF-RSC to deubiquitinate RIPK1 and suppress necroptosis^16^; (2) develop folate-, hyaluronic acid-, or antibody-conjugated targeted nanoparticles to specifically deliver *Abin-1* siRNA/miRNA to tumor sites^42^; and (3) Investigate small molecules capable of suppressing ABIN-1 expression. Using publicly available gene expression datasets with connectivity mapping, a commonly used approach to uncover novel medical indications for existing drugs may accelerate the search for ABIN-1 suppressors for clinical use. According to the COSMIC database, 7.14% CRC samples show ABIN-1 overexpression, while 6.04% CRC samples show ABIN-1 underexpression. Suppression of ABIN-1 may not be necessary in underexpressed CRC tissues, but in tumor tissues with normal or overexpression of ABIN-1, targeting ABIN-1 to enhance necroptosis-based therapy may be beneficial. Therefore, characterization of ABIN-1 expression in all cancer types sensitive to necroptosis is warranted.

In conclusion, ABIN-1 is a multiple cell death suppressor, and ABIN-1 deficiency breaks through the barrier of apoptosis and necroptosis in certain conditions. Moreover, necroptosis-based cancer therapy should be considered an alternative option to treat CRC patients with chemotherapeutic drug resistance. Targeting ABIN-1 will further improve this therapy to suppress CRC progression.

## Supplementary information

Supplementary Figure legends

Figure S1

Figure S2

Figure S3

Figure S4

Figure S5

## Supplementary information

**Supplementary information** The online version contains supplementary material available at 10.1038/s41419-021-03427-y.
