# Supplementary material for: ABIN-1 is a key regulator in RIPK1-dependent apoptosis (RDA) and necroptosis, and ABIN-1 deficiency potentiates necroptosis-based cancer therapy in colorectal cancer
Source: Cell Death Dis. 2021 Feb 1;12(2):140. doi: 10.1038/s41419-021-03427-y (PMC7862295; doi:10.1038/s41419-021-03427-y)
Supplement: Supplementary file 1 — Supplementary Figure legends [file 41419_2021_3427_MOESM1_ESM.doc]

**Supplementary Fig. S1**

**a** *Abin-1^+/+^* and *Abin-1^-/-^* MEFs were treated with cycloheximide (C), poly(I:C)+cycloheximide (PC), or P5 for 6h in presence or absence of Nec-1s. **b** *Abin-1^+/+^* and *Abin-1^-/-^*MEFs were treated with GSK872, poly(I:C)+zVAD (PZ), PZ+GSK-872, or PZ+GSK-872+Nec-1s for 7h and 24h. **c** ABIN-1 knockdown efficiency in *Tak1^+/+^* MEFs and *Tak1^-/-^* MEFs. **d, e** RIPK3 and MLKL knockdown efficiency in *Abin-1^+/+^* and *Abin-1^-/-^* MEFs. **f-g** Rig-I and TLR3 knockdown efficiency in *Abin-1^+/+^* and *Abin-1^-/-^*MEFs. **h, i** *Abin-1^+/+^*, *Abin-1^+/-^* and *Abin-1^-/-^* MEFs were transfected with NC or *Mda5* siRNA for 36h and then treated with P5 or P5Z for 6h. Cell deaths were measured by ToxiLight assay (**h**); qPCR assay of MDA5 knockdown efficiency (**i**). *p < 0.05, **p < 0.01, or ***p < 0.001.

**Supplementary Fig. S2**

**a, b** COLO205 NC shRNA cells and *Abin-1* shRNA cells were transfected with NC siRNA or *Tak1* siRNA and incubated for 24h. Then, cells were re-plated and treated with poly(I:C) (P) or poly(I:C)+5Z-7 (P5) for 24h. Cell deaths were measured by ToxiLight assay. Cell death in NC siRNA transfected cells (**a**); Cell death in *Tak1* siRNA transfected cells (**b**). **c** COLO205 NC shRNA and *Abin-1* shRNA cells were treated with P5 or P5I with or without Nec-1s, and then cells were lysed at indicated time points, followed by Western blot analysis of ABIN-1 and apoptosis markers cleaved caspase-3 and PARP-1 and necroptosis marker p-MLKL. *p < 0.05, **p < 0.01, or ***p < 0.001.

**Supplementary Fig. S3**

**a** HT-29 cells were transfected with control plasmid (pcDNA3.1-EGFP) or ABIN-1 plasmid (pcDNA3.1-ABIN-1-EGFP) and incubated for 24h. Then, cells were re-plated and treated with TBI for 8h in presence or absence of Nec-1s. **b** Western blot analysis of ectopic ABIN-1 expression and endogenous ABIN-1 expression. *p < 0.05, **p < 0.01, or ***p < 0.001.

**Supplementary Fig. S4**

COLO205 cells were transfected with NC siRNA or Abin-1 siRNA and incubated for 24h. Then, cells were re-plated and treated with Birinapant+IDN-6556 (BI) for 0-12h. Cell lysates were collected, and Western blot analysis of p-RIPK1, RIPK1, p-MLKL, MLKL, and ABIN-1 was performed.

**Supplementary Fig. S5**

Western blot analysis of necroptosis markers in tumor samples of Birinapant+IDN-6556 (BI) group (xenograft experiment in main figure 5). n=3.
