# Supplementary figures and images for: ABIN-1 is a key regulator in RIPK1-dependent apoptosis (RDA) and necroptosis, and ABIN-1 deficiency potentiates necroptosis-based cancer therapy in colorectal cancer
Source: Cell Death Dis. 2021 Feb 1;12(2):140. doi: 10.1038/s41419-021-03427-y (PMC7862295; doi:10.1038/s41419-021-03427-y)

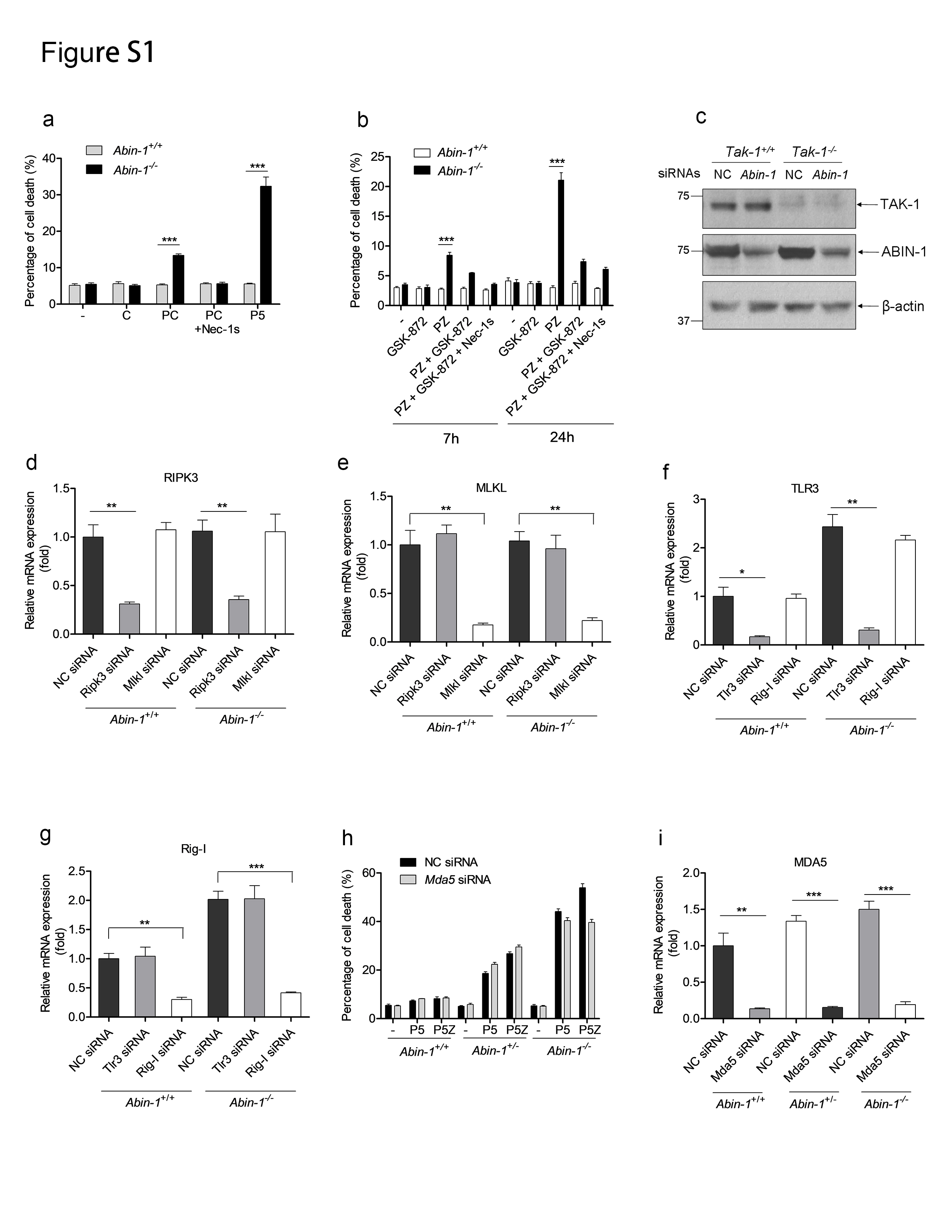

Supplement: Supplementary file 2 — Figure S1 [file 41419_2021_3427_MOESM2_ESM.jpg]

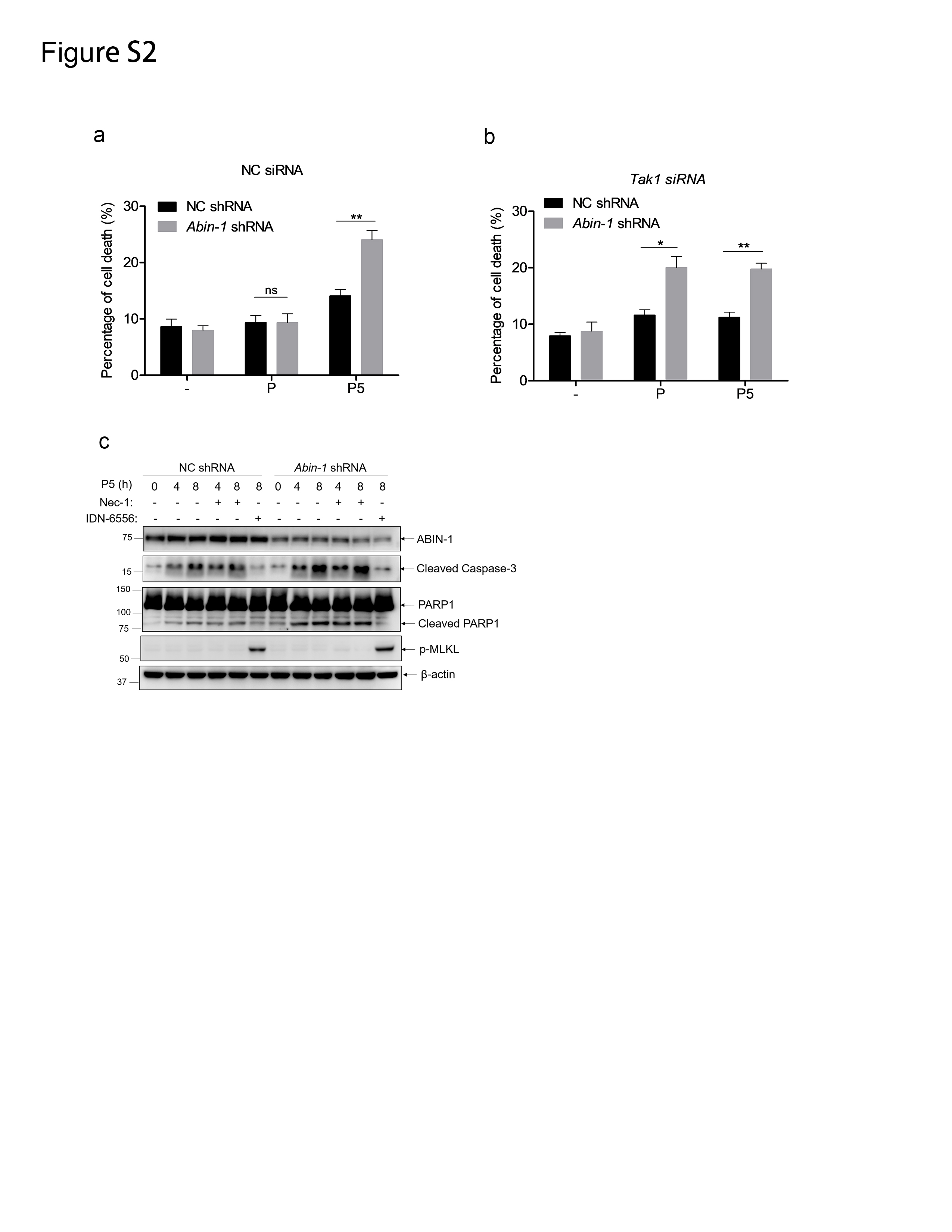

Supplement: Supplementary file 3 — Figure S2 [file 41419_2021_3427_MOESM3_ESM.jpg]

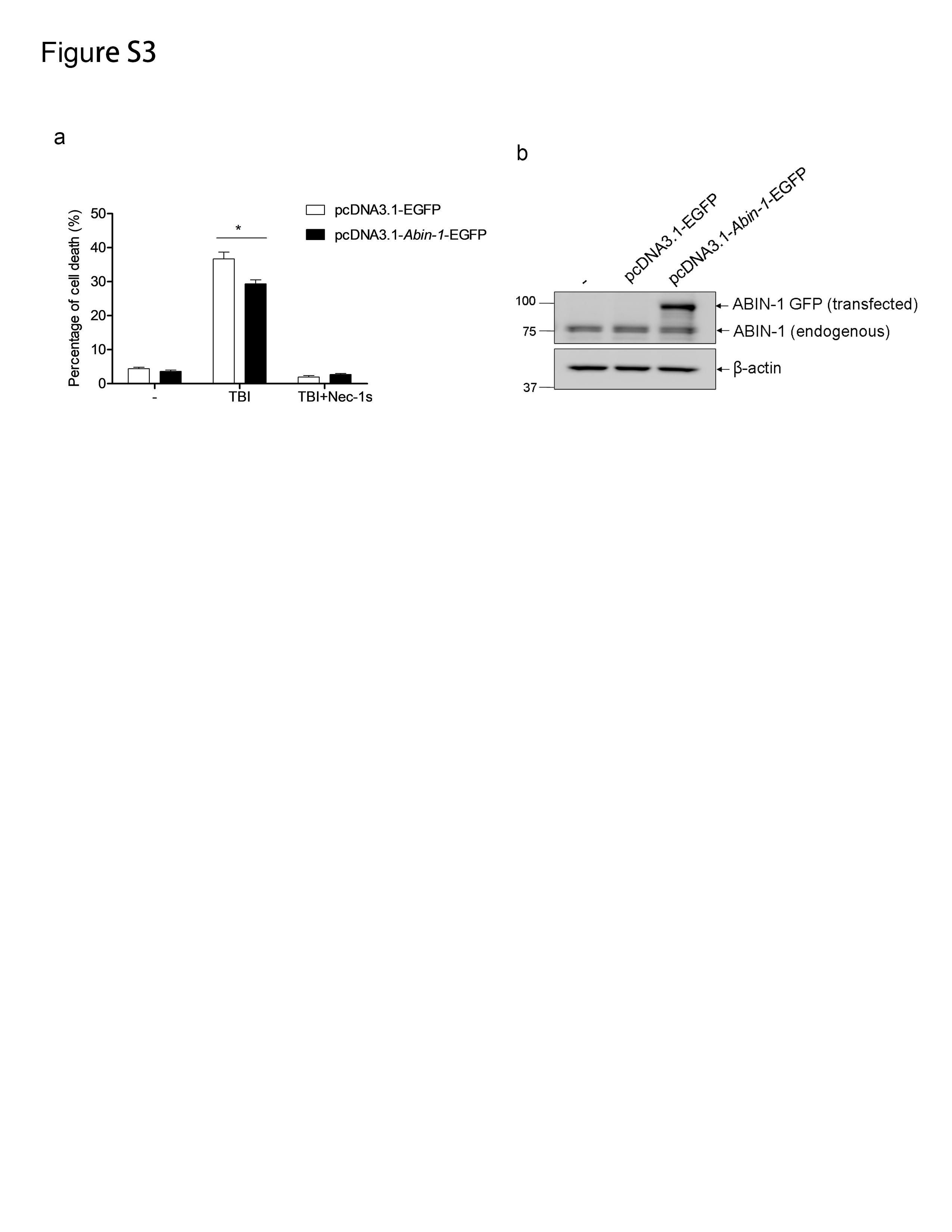

Supplement: Supplementary file 4 — Figure S3 [file 41419_2021_3427_MOESM4_ESM.jpg]

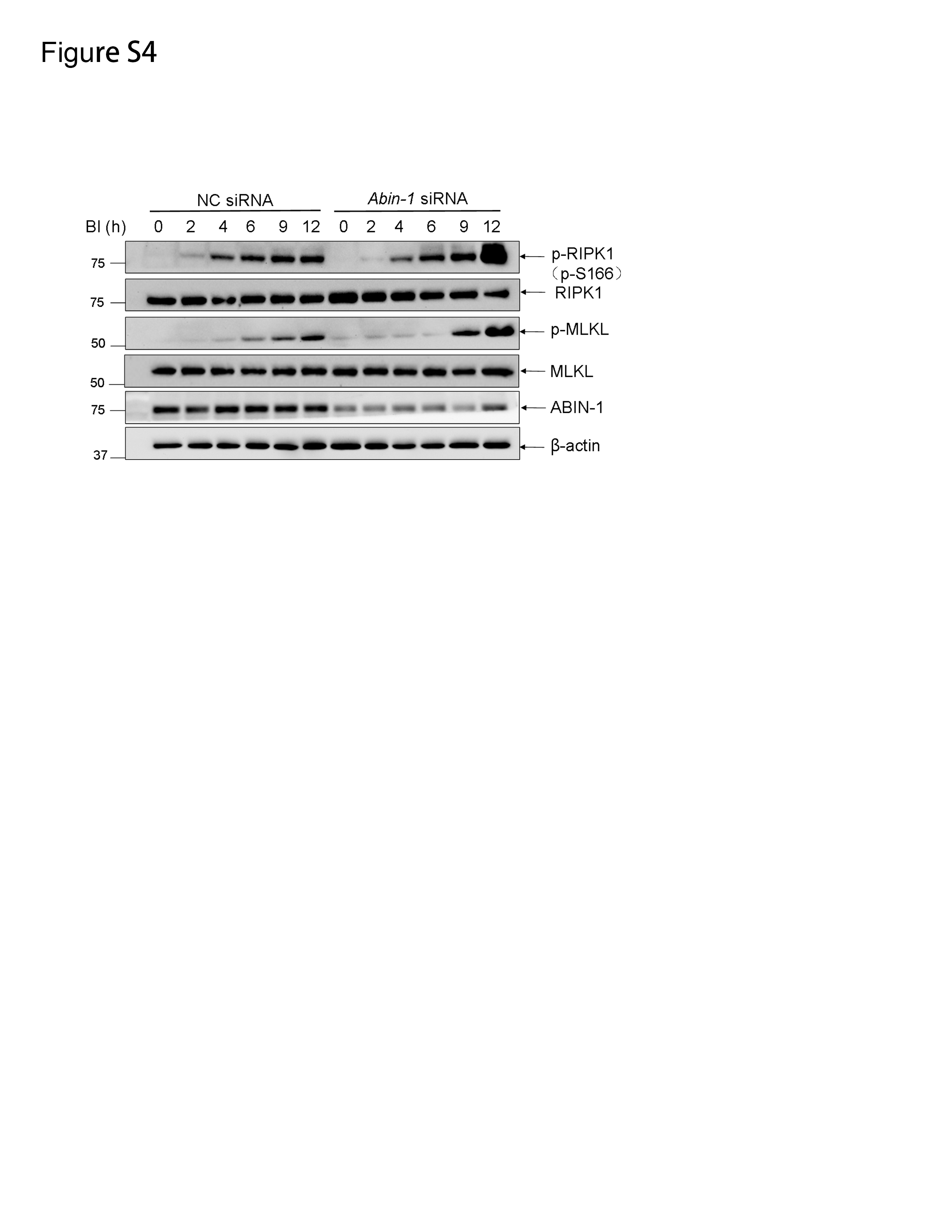

Supplement: Supplementary file 5 — Figure S4 [file 41419_2021_3427_MOESM5_ESM.jpg]

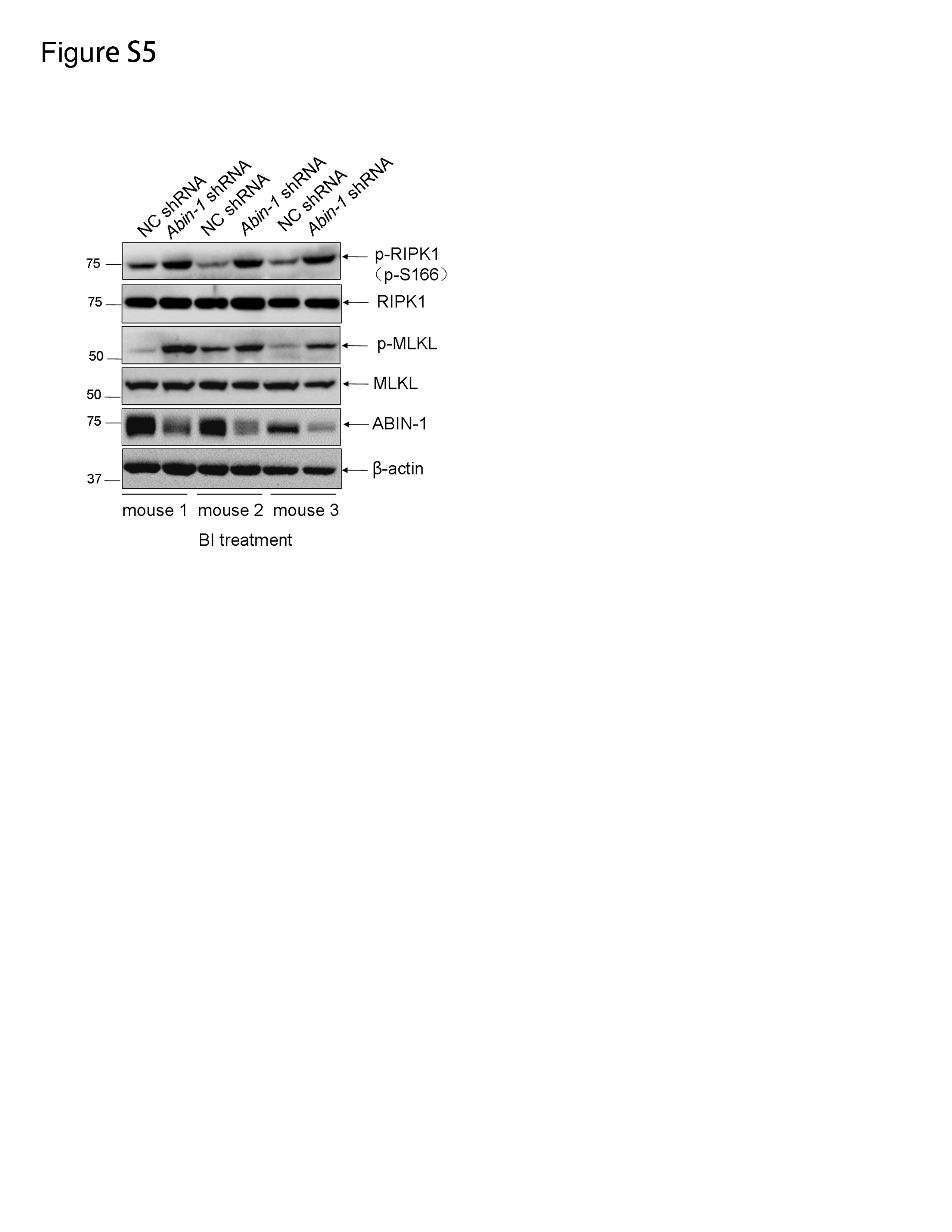

Supplement: Supplementary file 6 — Figure S5 [file 41419_2021_3427_MOESM6_ESM.jpg]
